# Armed to the teeth: The underestimated diversity in tooth shape in snakes and its relation to feeding behavior and diet

**DOI:** 10.1002/ece3.10011

**Published:** 2023-04-13

**Authors:** Marion Segall, Céline Houssin, Arnaud Delapré, Raphaël Cornette, Anthony Herrel, Joshua Milgram, Ron Shahar, Maïtena Dumont

**Affiliations:** ^1^ Department of Life Sciences The Natural History Museum London UK; ^2^ Institut de Systématique, Evolution, Biodiversité (ISYEB), UMR 7205 Muséum National d'Histoire naturelle, CNRS, SU, EPHE, UA Paris France; ^3^ Mécanismes Adaptatifs et Evolution, UMR 7179, Muséum National d'Histoire Naturelle CNRS Paris France; ^4^ Laboratory of Bone Biomechanics, Koret School of Veterinary Medicine The Robert H. Smith Faculty of Agriculture, Food and Environment, HUJI Rehovot Israel

**Keywords:** 3D geometric morphometrics, dental morphology, dentary teeth, ecomorphology, feeding ecology, snakes

## Abstract

The structure, composition, and shape of teeth have been related to dietary specialization in many vertebrate species, but comparative studies on snakes' teeth are lacking. Yet, snakes have diverse dietary habits that may impact the shape of their teeth. We hypothesize that prey properties, such as hardness and shape, as well as feeding behavior, such as aquatic or arboreal predation, or holding vigorous prey, impose constraints on the evolution of tooth shape in snakes. We compared the morphology of the dentary teeth of 63 species that cover the phylogenetic and dietary diversity of snakes, using 3D geometric morphometrics and linear measurements. Our results show that prey hardness, foraging substrate, and the main feeding mechanical challenge are important drivers of tooth shape, size, and curvature. Overall, long, slender, curved teeth with a thin layer of hard tissue are observed in species that need to maintain a grip on their prey. Short, stout, less curved teeth are associated with species that undergo high or repeated loads. Our study demonstrates the diversity of tooth morphology in snakes and the need to investigate its underlying functional implications to better understand the evolution of teeth in vertebrates.

## Graphical abstract

Among vertebrates, snakes have a unique feeding behavior during which their teeth fulfils several functions, which could have reduced their morphological diversity. Yet, our results show that the shape of dentary teeth in snakes is variable and is associated with feeding ecology and biomechanics.

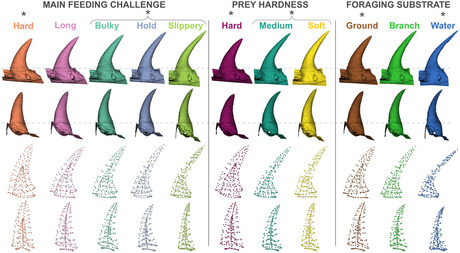

## 1 INTRODUCTION

Teeth allow most vertebrates to acquire food –and therefore energy—from their environment (Lucas, 2004). As such, they are one of the critical interfaces between an animal and its environment and their evolution depends both on intrinsic and extrinsic constraints. Teeth play different roles, from food acquisition to food processing, and have different functions (e.g., cutting, crushing, grinding, piercing). These functions depend on both food properties and/or food processing behavior and are related to tooth shape (e.g., Crofts et al., 2020). Work by Crofts et al. (2020), based on pioneering studies by Lucas (2004) and Massare (1987), showed how tooth morphology is associated with prey properties and dental biomechanics (e.g., toughness, bending abilities, mode of failure). Rounded teeth, for example, allow to crush hard prey items, they are tough, can barely bend, and consequently are susceptible to fragmentation (Crofts et al., 2020). Because of their tight and reliable relationship with diet and their abundance in the fossil record, teeth have been suggested to be good indicators of past climate and paleoenvironments (e.g., Evans, 2013), and are used to make inferences on the ecology of extinct species (Bellwood et al., 2014; Evans & Pineda‐Munoz, 2018; Fischer et al., 2022; Frederickson et al., 2018; Massare, 1987). By extension, tooth morphology could also be used to infer the feeding habits of secretive species that are sometimes only known from museum specimens, providing the link between tooth shape and food properties has been established. While most dental morphology studies have focused on mammals, because they benefit from a large variety of diets and tooth shapes (Berkovitz & Shellis, 2018; Ungar, 2010, 2015), a significant amount of work has been done on nonmammalian vertebrates (e.g., Clifton & Motta, 1998; Hotton, 1955; Linde et al., 2004; Montanucci, 1968; Presch, 1974; Rajabizadeh et al., 2020), (for a review: Berkovitz & Shellis, 2017). Yet, quantitative comparisons of tooth morphology and its link to dietary ecology, in a phylogenetically and ecologically broad sample of species remain rather scarce for nonmammalian vertebrates. In this study, we investigated the relationship between dietary ecology and tooth shape in a group of nonmodel vertebrates: snakes.

Among vertebrates, macrostomatan snakes are peculiar as they are the only taxon able to ingest prey larger than their head without processing it. This behavior is related to an extraordinary organization of the skull that has become highly kinetic. Indeed, snakes must coordinate the movements of eight pairs of cranial bones to catch, subdue, manipulate, and swallow their prey (Cundall & Greene, 2000; Moon et al., 2019). Despite the complexity of their feeding behavior, snakes have independently adopted a wide variety of dietary preferences (gastropods, mammals, birds, crustaceans) providing an opportunity to study possible convergences in their feeding apparatus (Rhoda et al., 2020). In addition to constraints related to the physical properties of their food items, some feeding behaviors may impose high loads on snake teeth, such as eating live and vigorous prey, with or without the support of a solid substrate. These various mechanical challenges may have driven the evolution of tooth shape in snakes. Despite their richness and complexity in shape (Vaeth et al., 1985; Young & Kardong, 1996), studies on snake tooth morphology are scarce and either lack a quantitative approach or are phylogenetically limited (Berkovitz & Shellis, 2017; Britt et al., 2009; Evans et al., 2019; Rajabizadeh et al., 2020; Ryerson & Van Valkenburgh, 2021). Fangs, and mostly front fangs, have recently attracted some scientific attention (Broeckhoven & du Plessis, 2017; Cleuren, Parker, et al., 2021; Crofts et al., 2019; Kundanati et al., 2020; Palci et al., 2021; Plessis et al., 2018). Yet, fangs are phylogenetically and functionally limited; their only purpose is to puncture the prey to deliver venom and consequently, fangs are not representative of snake tooth diversity. Indeed, they are but two highly derived teeth out of sometimes over 200 teeth (D. Rhoda pers. obs.). However, fangs may play an underestimated role in prey manipulation and swallowing, but their primary purpose is puncturing and venom injection.

Snake teeth are usually described as pointy and curved (Berkovitz & Shellis, 2017). They would therefore be considered as “piercing” specialists in the classification scheme as described in (Crofts et al., 2020), and should be associated with a restricted diet composed of soft invertebrates and small fish. Yet, as previously mentioned, snakes show a broad variety of diets but also a wide variety of feeding behaviors that involve their teeth such as “chewing” (Tumlison & Roberts, 2018), ripping (Bringsøe et al., 2020; Jayne et al., 2002; Noonloy et al., 2018), slicing (Cundall & Greene, 2000; Kojima et al., 2020), or swallowing without piercing (e.g., *Dasypeltis sp*.). Snake teeth are also involved in the whole feeding sequence, from prey capture to swallowing (Cundall & Greene, 2000; Deufel & Cundall, 2003b; Ryerson & Van Valkenburgh, 2021). Yet, the diversity of tooth morphology and function in snakes remains under‐explored. Here, we quantified and compared the dentary tooth morphology of 63 species that cover the phylogenetic and ecological breadth of snakes. We tested four factors related to feeding that could be associated with morphological adaptations of the teeth:

- Prey hardness: Prey hardness is related to tooth shape in other vertebrates (Berkovitz & Shellis, 2017, 2018). Teeth of durophagous species are usually more blunt, and their shape is adapted to resist high loads while crushing a prey (Crofts, 2015). Snakes do not crush their prey (except the crustacean specialist *Fordonia leucobalia*), but they use their teeth to manipulate and swallow it whole. Their teeth must resist high loads –albeit lower than other crushing‐durophagous animals. Durophagy has been associated with short and blunt fangs (Cleuren, Hocking, & Evans, 2021) and dentary teeth (in a study comparing four closely related species: Rajabizadeh et al., 2020), while species feeding on soft prey have sharp and long fangs whole (Cleuren, Hocking, & Evans, 2021). We expect dentary teeth to present similar morphological adaptations to prey hardness as fangs.
- Prey shape: Snakes are vulnerable to predators during the manipulation and swallowing of prey and must reduce the time and energy spent during feeding (Arnold, 1993). The shape of the prey eaten involves different biomechanical challenges for snakes (Vincent et al., 2006) and is associated with different head shapes (Segall et al., 2020). Bulky prey (e.g., anurans, mammals) require extensive manipulation (Pough & Groves, 1983) and repositioning while long prey (e.g., snakes, eels) require more “pterygoid walks” (i.e., protractions and retractions of left and right tooth rows in alternating fashion assuring intraoral transport). Manipulation and repositioning of bulky prey require a good grip, which can be provided by long, curved, and sharp teeth. While having short teeth that are not easily embedded in the prey at each pterygoid walk may allow to reduce the swallowing duration when eating elongated prey.
- Foraging substrate: Feeding on the ground provides a solid substrate for the snake to support either itself or the prey during capture, subduction, manipulation, and swallowing (Moon et al., 2019). However, feeding in an aquatic or arboreal context does not provide that support, so we expect the teeth to play a larger role in those ecological contexts. Aquatic snakes must deal with their own buoyancy and that of their prey while feeding, so the forces applying on the teeth may show a diversity of orientations. We expect the teeth of aquatic species to have a shape that allows some degree of bending. Arboreal manipulation and swallowing usually happen with the snake hanging from a branch, head down, with no substrate support and almost without the help of the rest of its body. This behavior increases the chances of dropping prey, which may not be possible for the snake to retrieve, unlike in an aquatic or terrestrial environment. Thus, arboreal feeding requires a good grip on the prey, which would be favored by long, sharp, and curved teeth.
- Biomechanical challenge related to feeding ecology: The previous factors are not mutually exclusive (e.g., eel‐eaters eat elongated prey underwater) but are also restrictive as they do not account for feeding behavior. For instance, terrestrial viperids can either envenomate and release their prey, while others hold on to the prey after striking (Glaudas et al., 2017). The first behavior involves manipulating and swallowing a dead prey, while the prey is alive and certainly fighting back in the second case. These two behaviors certainly impose different loads on the teeth. We classified characteristics associated with the prey and the feeding behavior that impose mechanical challenges to the teeth. We consider that (1) hard prey is the highest challenge for a tooth, followed by (2) elongated prey that involve repeated loading, (3) slippery prey (secreting mucus) that may impose loads in various directions and require a good grip to prevent escape, (4) Holding a vigorous prey that is neither hard, nor long, nor slippery, finally, (5) bulky prey that requires extensive tooth manipulation.

We dissected the dentary bone of 63 species of snakes and used micro‐CT scanning to obtain high‐resolution scans of the teeth. We then used 3D geometric morphometrics to compare both the external and internal shapes of the teeth. Shape information on the inner part of the teeth allowed us to compare the thickness of the hard tissues that compose the tooth. We also measured the length and maximal and average degrees of curvature. Next, we used phylogenetic comparative methods to test the importance of our predictive ecological factors as drivers of tooth shape to establish the link between tooth shape variation and dietary ecology in snakes.

## 2 MATERIALS AND METHODS

### 2.1 Tooth shape acquisition

We dissected 63 species that cover both the phylogenetic and dietary diversity of Alethinophidians (Datas S1 and S2). Our sample is composed of adult or subadult specimens from museums (AMNH, MNHN, Jerusalem University) and private collections (details in Datas S1 and S2). While comparisons of the shape of the teeth from different bones show a significant ecological signal in some clades (Fischer et al., 2022) we here decided to focus only on the dentary bone to ensure functional homology. In snakes, the dentary is involved in capturing, restraining, and manipulating the prey; so, it endures loads related to both prey characteristics (e.g., hardness) and feeding behavior (e.g., holding vigorous prey, extracting a snail from its shell). The maxilla has a similar function, but it is too functionally and morphologically derived in venomous snakes to be able to make a fair comparison. We isolated the right dentary bone of the specimens and CT scanned it using a Phoenix Nanotom S μCT‐scanner (General Electric) at the Institut de Genomique Fonctionelle, Ecole Normale Superieure. For *Crotalus adamanteus*, *Eryx jaculus*, and *Dasypeltis scabra*, we scanned the left dentary and mirrored the mesh. The bones were scanned at mid‐section to ensure homology of position in the teeth and as close as possible to the teeth to get the highest possible resolution (voxel size between 0.97–7.50 μm), with a voltage of 100 kV and a current of 70 μA. Only the *Python regius* tooth was not a mid‐section but the first tooth of the dentary (but removing it from the sample barely changed the statistical results). The 3D reconstruction was done using Phoenix datos|x2 (v2.3.0, General Electric) and the subsequent segmentation was performed using VGStudioMax (v1.0, Volume Graphics GmbH). We segmented teeth that were not broken or in the process of being replaced, which was easily noticeable on the scans through resorption of the bony attachment in most species. We obtained one tooth per specimen (63 dentary teeth) positioned in the middle section of the dentary to ensure homology in position and function, the only exception was *Python regius*. Comparisons between single teeth (from different bones) in phylogenetically diverse samples have demonstrated an ecological signal (Fischer et al., 2022), and the interspecific shape variation is larger than the intraspecific variation in our sample, validating the use of one tooth per species (see Data S2b).

**FIGURE 1:**
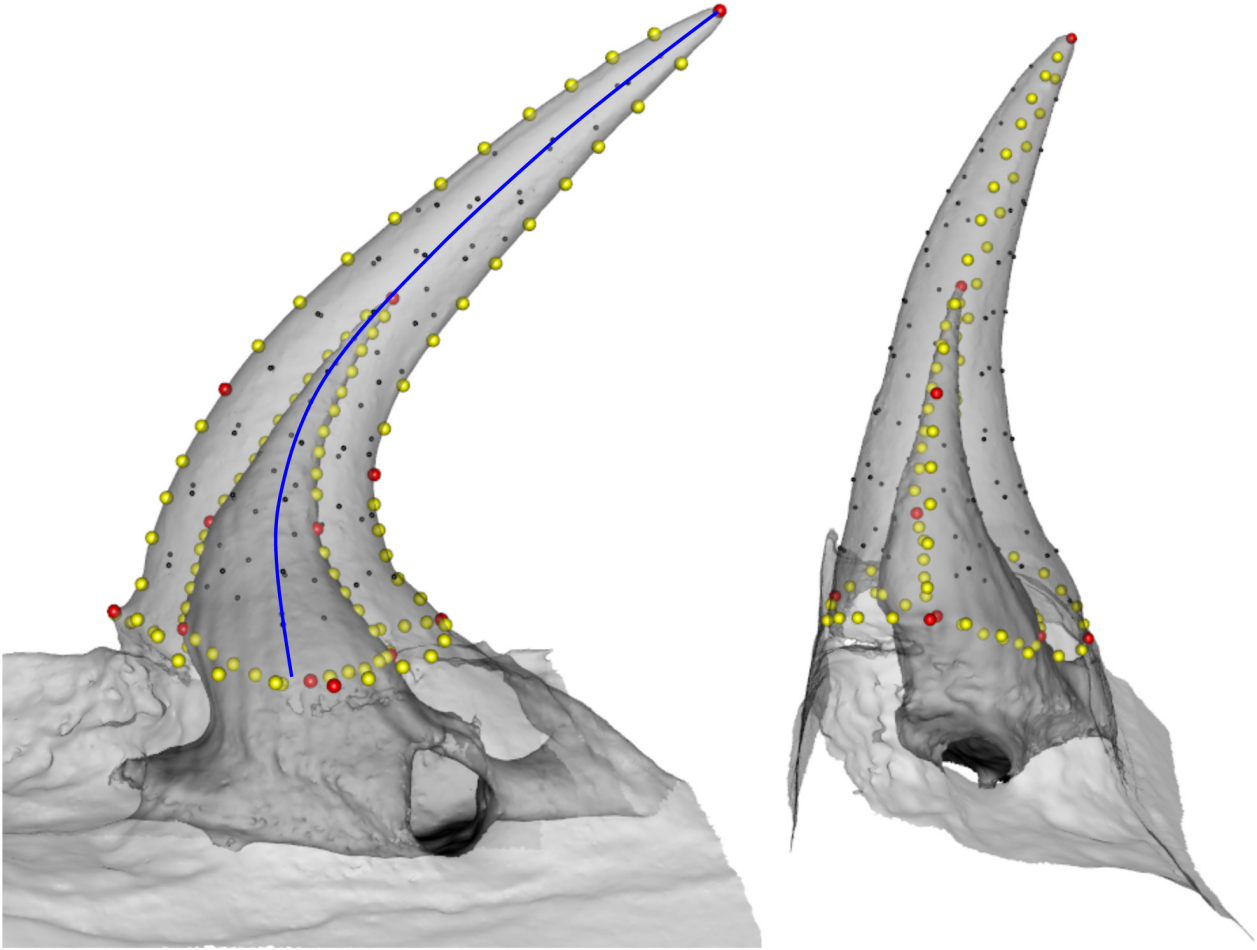
Snake tooth template illustrating the landmarks used for geometric morphometric analysis. 3D mesh of a dentary tooth of Natrix tessellata (HUJI 16537) in medial (left) and anterior (right) view. The scan is semi‐transparent to show the inner layer of the tooth. Landmarks are represented by spheres colored in red for anatomical landmarks, yellow for curve semi‐landmarks, and black for surface semi‐landmarks. A total of 221 landmarks were used to describe the 3D shape of snake teeth. The blue line is the midline of the tooth, its length is called L_C_ and its mean and maximum of curvature (κ_mean_, κ_max_) were obtained using the kappa plugin.

We used 3D geometric morphometrics to quantify the 3D shape of the teeth. We placed 14 anatomical landmarks: 7 on the outer surface of the teeth, 7 on the pulp cavity surface (inner part of the tooth Data S3), and 100 curve semi‐landmarks (50 on each layer) using the software MorphoDig 1.2 (Lebrun, 2017). Curves correspond to the anterior and posterior edges of both layers and to the limit of the tooth insertion onto the bone. In addition, 42 and 65 surface semi‐landmarks were, respectively, placed on the inner and outer surfaces of the teeth to obtain an accurate 3D representation of the tooth shape (Figure 1). This template allows us to obtain information both on their shape and on the thickness of the hard tissue material (dentine and enamel). We placed the anatomical landmarks and curve semi‐landmarks by hand on each specimen and checked for the repeatability of the positioning by digitizing these six times in five specimens for which the teeth looked similar. A Principal Component Analysis demonstrated the repeatability of the positioning of our 14 anatomical landmarks and the necessity of using curve and surface semi‐landmarks (Data S4). We used the “Morpho” package (Schlager, 2015) to project and relax the surface semi‐landmarks on each specimen and to slide the curve and surface semi‐landmarks while minimizing the bending energy between the specimen and the template (Gunz & Mitteroecker, 2013). We then performed a Procrustes superimposition using the function *gpagen* of the “geomorph” package (Adams et al., 2020), and the resulting Procrustes coordinates were used to test our hypotheses. The projection, relaxation, and Procrustes superimposition were performed using R version 3.4.4 (R Core Team, 2018).

As 3D geometric morphometrics removes size information, we also took linear and angle measurements of the teeth to test their potential link with dietary ecology. We measured the length of the curvature of the tooth (L_C,_ Figure 1) along with the maximal and mean curvature (κ_mean_, κ_max_) of each tooth using the FIJI (v1.53q) plugin “Kappa” (Mary & Brouhard, 2019). To do so, we used a snapshot of the medial side of each tooth, obtained in GeomagicStudio 2013 (3D Systems, Rock Hill). We digitized the midline (Figure 1) to obtain its global curvature (see examples in Data S5, raw measurements available in Data S1). The plugin creates a series of point along the digitized curve, calculates the curvature at each point, and provides the maxima of curvature (κ_max_) and the average of all the curvature measurements (κ_mean_). The curvature is the inverse of the radius of the circle that would fit the curve at a specific point and is not, in our opinion, the most intuitive biological measure. We therefore converted these two curvature measurements into angles (degrees of curvature) using the following equation:

(1) $$D_{C}=\left(\;\kappa \times L_{C}\;\right)\;\mathrm{mod}\;2\pi \times \frac{180}{\pi },$$

From this equation, we obtained the mean and max curvature degrees of curvature (*D*_Cmean,_ *D*_Cmax_), the larger the angle, the curvier the line.

### 2.2 Dietary factors

An extensive bibliographic study was performed to characterize the diet, feeding behavior, and ecology of the 63 species (Data S1). Following our hypotheses, we defined four factors that could impact the shape of the teeth in snakes: prey hardness, prey shape, feeding substrate, and main mechanical challenge. We defined the three levels of prey hardness: soft (e.g., gastropods, annelids, birds, mammals), medium (e.g., amphibians, fishes, thin‐scaled lizards such as anoles), and hard (insects, crustaceans, snakes, hard‐scaled lizards such as skinks, Savitzky, 1983). Prey shape has two levels: bulky or long following descriptions by Segall et al. (2020). Mammals are considered bulky as the hind limbs are particularly difficult to swallow and require extensive manipulation in snakes. For generalist snakes that do not show a preferred prey, we did not attribute them to any prey shape group (“na” in Data S1). The foraging substrate has three levels: ground, water, and branch depending on where the swallowing of the food occurs. The main mechanical challenge encountered by snakes while feeding has five levels: hard (chitinous preys, hard‐scaled lizards), long (snakes, soft‐scaled lizards, earthworms), hold (snakes that maintain vigorous preys), slippery (fish, snails, amphibian eggs), bulky (mammals, amphibians).

### 2.3 Analyses

We used phylogenetic comparative methods, using a tree pruned from Pyron and Burbrink (2014). If species were not present in this tree, we used the closest relative (Data S2). We tested whether tooth curvature length, the mean degree of curvature, and the maximum degree of curvature were correlated with one another using Pearson correlation tests (function *cor.test* from the “stats” package). We then tested whether these measurements were associated with our factors using the function *phylANOVA* of the package “phytools” using 1000 simulations and a Holm correction for the post‐hoc pairwise *t*‐tests. The normality of the data distribution was checked using a Shapiro test, and variables were log‐transformed to ensure normality when necessary. We used the function *procD.pgls* in “geomorph” (Adams, 2014) to run phylogenetic ANCOVAs and ANOVAs to test the link between tooth shape and our different dietary constraints. For ANCOVAs, we used the log‐transformed centroid size of the tooth as a covariate. Since our predictive factors are not entirely independent from one another, we performed one model per factor. We compared the fit of our different models using the function *model. comparison* in the “RRPP” package (Collyer & Adams, 2022), which calculated the log‐likelihood of each model. Statistical significance was tested by performing 10,000 permutations. We performed subsequent post‐hoc pairwise comparisons using the *pairwise* function in “RRPP” on the best models. All geometric morphometric, statistical analyses, and visualizations were performed in R version 3.4.4 (R Core Team, 2018) (R code and data available in Supplementary Material), except the landmark acquisition performed in MorphoDig (Lebrun, 2017). To highlight shape differences between ecological groups, we used mesh deformation of the template specimen toward the mean landmark configuration for each group, using the *plotRefToTarget* function of the “geomorph” package. We also used the function *spheres3d* from the “rgl” package that allows us to visualize both the inner and outer surfaces of the teeth, providing information on teeth thickness (Figure 5).

## 3 RESULTS

### 3.1 Tooth length, curvatures, and feeding ecology

**FIGURE 2:**
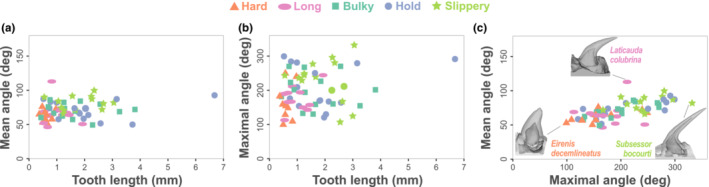
Correlations between linear measurements of the tooth. (a) Tooth length and mean angle, (b) tooth length and maximal angle, (c) mean angle and maximal angle. Each dot represents one species and is colored and shaped according to the main mechanical challenge encountered during feeding (legend above the graph).

**FIGURE 3:**
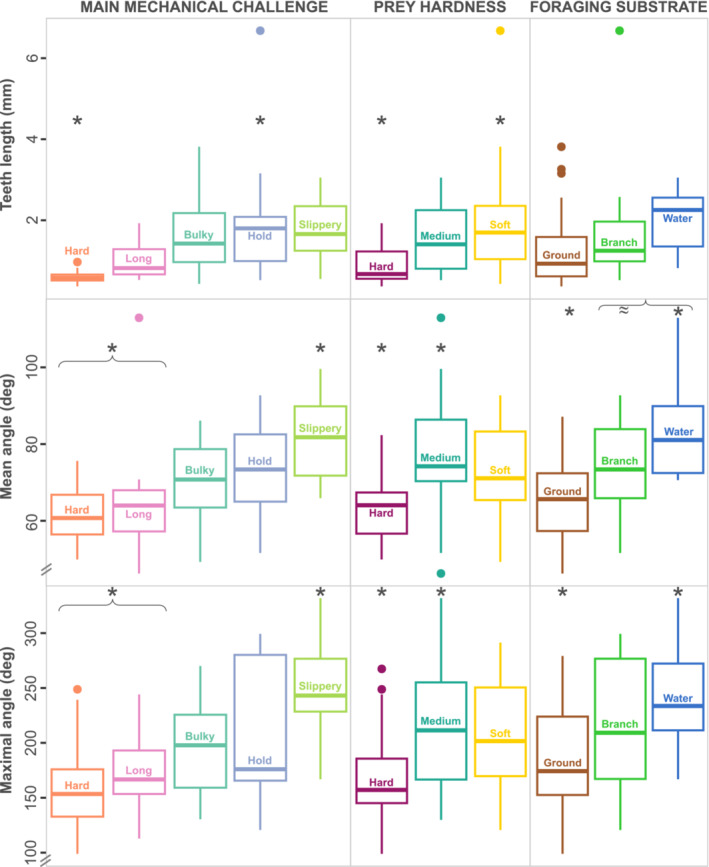
Boxplots representing the differences in tooth length (top), mean angle (middle), and max angle (bottom) depending on feeding ecological factors. First column: differences related to the main mechanical challenge; second column: prey hardness; and third column: foraging substrate. Statistically significant differences are indicated by *. Groups in the same bracket are not significantly different but are different from groups with the asterisk (e.g., mean angle is different between hard and slippery, and long and slippery). ≈ indicates a nearly significant result.

Dentary tooth length (*L*_C_) in our sample varies 18‐fold (0.37–6.68 mm), with a mean of around 1.2 mm. The average curvature (mean angle) of the teeth varies between 46–113°, with a median of around 70.6°. The maximal degree of curvature varies between 99–332° with a median of around 194° (Figures 2 and 3). We found significant, yet weak, positive correlations between teeth length and the mean (*p* = .03, *R* = 0.26) and max degrees of curvature (*p* = .03, *R* = 0.27). The correlation between the mean and max degrees of curvature is also significant; snakes with a higher mean curvature also have a high maximal angle of curvature (*p* < .0001, *R* = 0.64).

The main mechanical feeding challenge is significantly related to the three measurements (*L*_C_: *F* = 4.03, *p* = .03, mean angle: *F* = 4.49, *p* = .02, max angle: *F* = 5.06, *p* = .01). Pairwise comparisons show that snakes that must hold their prey have longer teeth than snakes feeding on hard prey and snakes feeding on slippery prey, which both have a larger mean and max. angle of curvature than snakes feeding on hard and long prey (Figure 3, Table 1).

**TABLE 1:** Summary of the significant pairwise tests. Italic indicates a nearly significant result.

	MAIN MECHANICAL CHALLENGE			
	Hard ‐ hold	Hard ‐ slippery	Hard ‐ bulky	Slippery ‐ long
Length	*t* = 3.32, *p* = .04			
Mean curvature		*t* = 3.87, *p* = .06		*t* = 3.17, *p* = .03
Max curvature		*t* = 4.01, *p* = .007		*t* = 3.47, *p* = .01
Shape	*d* = 0.03, *p* = .004	*d* = 0.04, *p* < .001	*d* = 0.03, *p* = .006	

	PREY HARDNESS			
	hard ‐ soft		hard ‐ medium	
Length	*t* = 3.42, *p* = .02			
Mean curvature			*t* = 3.53, *p* = .002	
Max curvature			*t* = 2.95, *p* = .01	
Shape	*d* = 0.023, *p* = .01		*d* = 0.024, *p* = .01	

	FORAGING SUBSTRATE			
	ground ‐ branch		ground ‐ water	
Mean curvature	*t = −2.34*, *p = .052*		*t* = 4.28, *p* = .0006	
Max curvature			*t* = 3.02, *p* = .04	
Shape	*d* = 0.024, *p* = .029			

Prey hardness is also related to tooth length (*F* = 5.93, *p* = .01), mean curvature (*F* = 6.38, *p* = .01), and the maximal angle of curvature (*F* = 4.93, *p* = .03). Snakes feeding on hard prey have shorter teeth than snakes preying upon soft prey. Snakes feeding on the prey of intermediate hardness have more curved teeth than durophagous species (Figure 3, Table 1).

The medium in which species feed is also significantly related to the mean (*F* = 9.97, *p* = .001) and maxima of curvature (*F* = 5.14, *p* = .03) but not to tooth length (*F* = 2.61, *p* = .15). Snakes that forage underwater have more curved teeth than snakes feeding on the ground. The average curvature of snakes feeding in trees is almost significantly higher than terrestrial species (*t* = −2.34, *p* = .052, Figure 3).

Finally, prey shape is not significantly related to the length and curvature (all *p* > .3).

### 3.2 Tooth shape and feeding ecology

**FIGURE 4:**
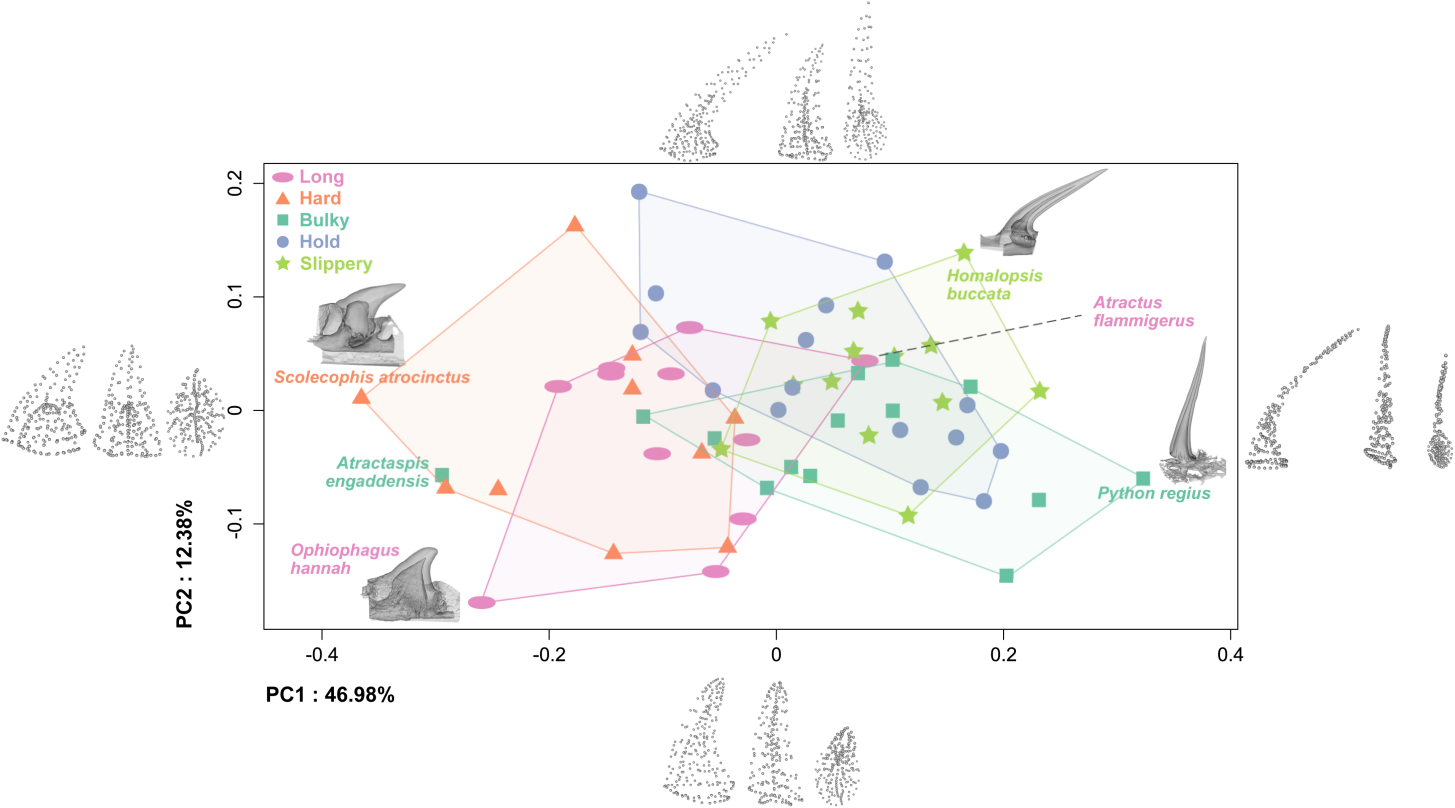
Tooth shape variability in snakes. Plot of the two first principal components that represent almost 60% of the shape variation. On the extreme of each PC, axis is represented the associated landmark configuration in (from left to right) medial, anterior, and top view. Four examples of scans are positioned in the morphological space to highlight shape diversity. Each dot represents one species and is colored and shaped according to their main feeding constraint.

There is a large variation in the shape of the teeth. The first principal component (PC1) that represents almost half of the variation in our sample (46.98%) differentiates very thin, slender teeth with a pulp cavity that runs along the full length of the tooth from short, stout teeth with a thick layer of tissue (Figure 4). PC2 differentiates teeth with a large base and a large maximal angle of curvature but not necessarily curved all along, from short, stout teeth that are mildly curved along their length. The distribution of species in the morphospace is not structured by phylogeny (Data S6). Species that eat long or hard prey are mostly positioned on the left side of the plot. Species that eat bulky, slippery prey, and species that must hold their prey are mostly positioned on the right side.

**FIGURE 5:**
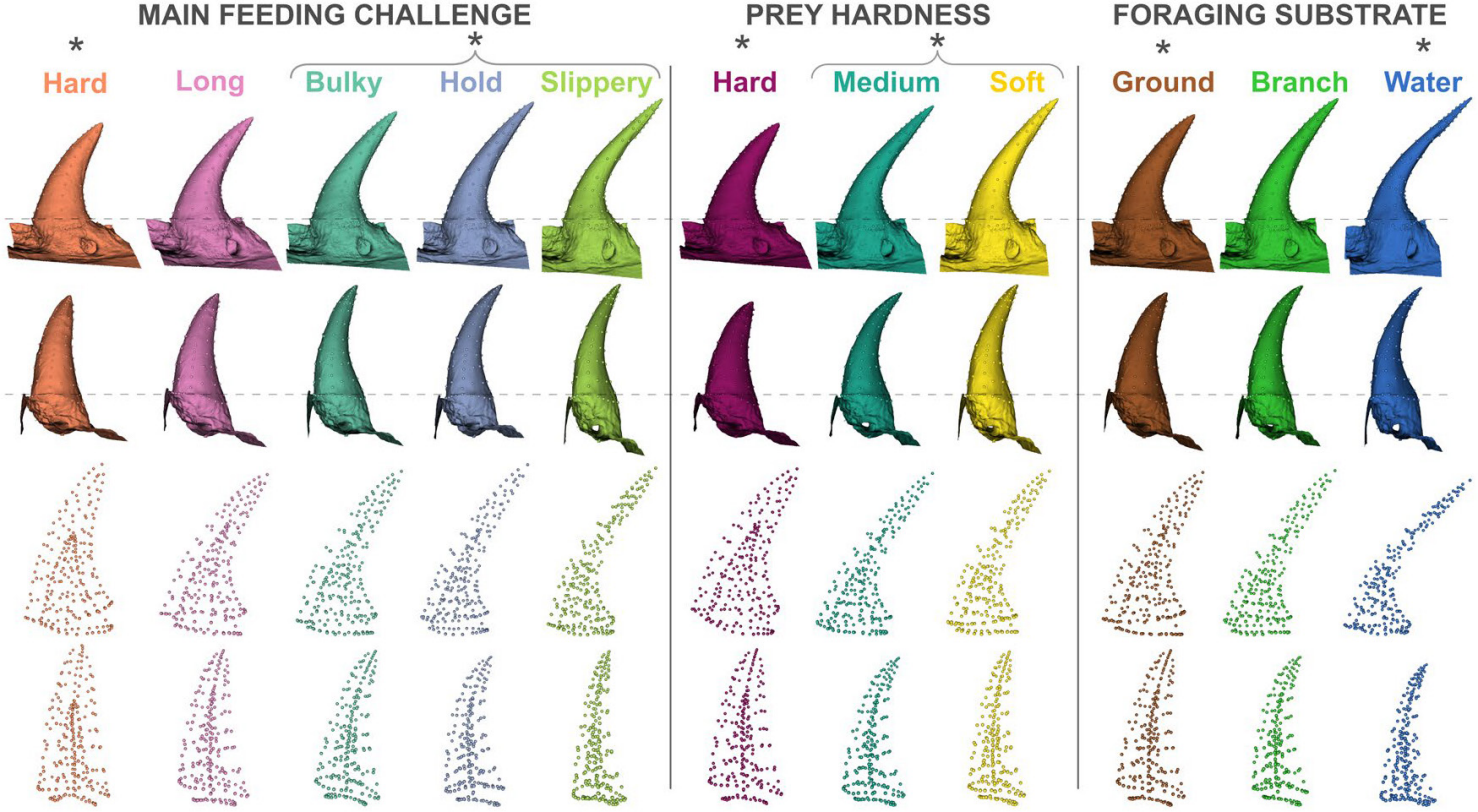
Mean tooth shape associated with significant ecological predictors. The first line shows the medial side of the teeth and the second line the anterior view. Meshes are aligned using anatomical landmarks (see dashed lines). The third and fourth lines show landmark configurations using spheres. Statistically significant differences are indicated using brackets: groups in the bracket are not significantly different but are different from groups with the asterisk (e.g., bulky, hold, slippery different from hard).

The linear models, with and without accounting for size, show similar results: the main mechanical challenge (size: *F* = 1.78, *p* = .001, no size: *F* = 3.13, *p* = .0001), prey hardness (size: *F* = 2.94, *p* = .0002, no size: *F* = 4.48, *p* = .0001) and foraging substrate (size: *F* = 2.24, *p* = .002, no size: *F* = 3.8, *p* = .0001) are significantly related to tooth shape, but prey shape is not (all *p* > .6). For each factor, the best models are the ones not accounting for size (Data S7). The post‐hoc pairwise comparisons show significant differences in tooth shape between snakes eating hard versus slippery and bulky prey and species that must hold their prey (Table 1). Tooth shape also differs between snakes feeding on hard prey and those feeding on softer prey (Table 1). Durophagous specialists have short, stout, barely medially curved teeth. Their posterior curvature is regular but not high. Their pulp cavity is short, so they have a relatively thicker layer of hard material (dentine and enamel). Teeth of species feeding on bulky, slippery, medium, soft prey and that hold their prey are long and slender, with a long pulp cavity (i.e., relatively less hard material). They are medially curved and show an elbow‐like curvature near the base of the tooth (Figure 5).

Terrestrially feeding species have short and stout teeth but are relatively more slender than durophagous species. Aquatically feeding snakes, however, have highly curved teeth, in both the medial and posterior directions. They also show an elbow‐like shape but compared with species feeding on slippery prey they have very thin, needle‐like tooth tips (Figure 5).

Prey shape is not significantly associated with tooth shape in snakes.

## 4 DISCUSSION

### 4.1 Gross morphology, length, curvature

From the sharp, elongated, and nearly straight teeth of the ball python (*Python regius*, Figure 4), to the round, bulky, and nearly flat teeth of the king cobra (*Ophiophagus hannah*; Figure 4), the morphological variability of dentary teeth in snakes is far larger than what transpires in the literature. Yet, snake teeth remain easily identifiable among vertebrates as they share some characteristics: they are all conical, with various degrees of sharpness, and –although to different degrees— posteriorly and medially curved (Figures 2, 3, 4, 5). Variation in our curvature measurements appears to be driven by both intrinsic and extrinsic factors. The mean and maximal degree of curvature are significantly but weakly related to tooth length (Figure 2). They are positively correlated with one another; it was not obvious that this relationship existed as the overall curvature can either be caused by an elbow‐like shape (high maxima of curvature, e.g., *Homalopsis buccata*, Figure 4) or a continuous curvature (high mean curvature, e.g., *Laticauda colubrina*, Figure 2). Yet, our results show that more curved teeth also have a larger maximal angle of curvature and overall straighter teeth have a smaller maximal angle of curvature. A posterior tooth curvature may reduce the risk of breakage when the snake strikes at high velocity and acceleration (Ryerson & Van Valkenburgh, 2021). An increase in the curvature is associated with a decrease in the maximal stress undergone by the teeth when out‐of‐plane forces are involved, which is likely to be the case during a feeding sequence. The curvature may also allow the prey to slide over the teeth into the mouth and to be impaled if moving backwards, thus preventing a potential escape.

### 4.2 Prey hardness

As predicted, snakes eating hard prey have short, stout, blunt, and less posteriorly and medially curved teeth, contrary to softer prey eaters that have long, slender, and more curved teeth (Figures 3 and 5). Compared with slender teeth, short and stout teeth undergo relatively smaller maximal stress coming from the compression force and the stress is concentrated at the tip of the tooth (Bar‐On, 2019; Rajabizadeh et al., 2020). This may reduce the risk of failure of the tooth while feeding on hard items but may cause fragmentation and wear of the tip. We noticed some tooth fragmentation in species such as *Fordonia leucobalia*. *Fordonia* is a peculiar species that feeds on crustaceans that are crushed and dismembered (Jayne et al., 2018). This behavior likely imposes a high load on the teeth, thus aggravating the fragmentation. Most durophagous snakes do not use their teeth to dismember their prey or to crush them like other vertebrates. They use their teeth to manipulate and swallow their prey (sometimes alive Arsovski et al., 2014). Consequently, they may not need to resist loads as high as other vertebrates. In fact, durophagous snakes generally do not pierce their prey, they only have superficial, but repeated contacts with it during capture and manipulation. These snakes may have evolved short and blunt teeth to limit fracture during capture and manipulation, but this tooth shape seems to be related to high prey escape rate, and the necessity for behavioral adjustments, such as coiling the body around the prey to limit losing the prey (Gripshover & Jayne, 2021). Some durophagous snakes have independently evolved hinged teeth that fold back when they swallow the prey but may rise if the prey moves backwards, a strategy preventing escape as well (Jackson et al., 1999; Savitzky, 1981). Some legless lizards specialized on eating hard‐scaled lizards show a similar specialization suggesting a convergent evolution of hard prey specialists in squamates (Patchell & Shine, 1986). On the other hand, the dentary teeth of snakes feeding on softer prey show morphological adaptations related to piercing and getting a grip on prey and preventing escape, such as an elbow‐like configuration and a sharp tip.

### 4.3 Foraging substrate

We hypothesized that snakes feeding on the ground benefit from the support of a solid substrate to capture, manipulate, and swallow their prey, while arboreal species must deal with gravity and the weight of their body and prey. Aquatic snakes, on the other hand, need to deal with prey buoyancy. Our results indicate a significant difference in tooth shape between terrestrial and aquatic feeders but not in tooth length. Aquatic snakes have an elbow‐like tooth (Figure 4 *Homalopsis buccata*, Figure 5: *Laticauda colubrina*, *Subsessor bocourti*), that is highly medially curved, with a very thin and sharp tip that may be advantageous to prevent prey escape (Figures 3 and 5). Their pulp cavity runs almost to the tip of the tooth, so the layer of hard tissue is smaller in those teeth, which may provide them with more flexibility than terrestrial species. Terrestrial species usually coil around their prey, thus restricting their movement, but aquatic snakes usually do not restrain their prey. This may impose forces on the teeth coming from all directions. Thus, having more flexible teeth may be advantageous for aquatic species. This hypothesis remains to be tested, but the teeth of aquatically feeding snakes are weakly ankylosed (Savitzky, 1983), suggesting another adaptation to prevent the failure of these slender and sharp teeth (Patchell & Shine, 1986).

### 4.4 Main mechanical challenge

The results of the analyses focusing on the main mechanical challenge show that the teeth of durophagous species are different from those of species that need extensive manipulation of their prey (bulky, hard, hold). The hard prey group is characterized by teeth that are short and stout, with the smallest mean and maximal curvature (Figures 3 and 5). Their pulp cavity is the short and the relative thickness of hard tissue is greater than for the other groups, making their teeth more robust. On the opposite, slippery prey eaters are characterized by long, slender, and highly curved teeth (Figures 3 and 5). Their pulp cavity is long and provides the teeth with a relatively thin layer of hard tissue, which may allow more bending, but this hypothesis remains to be tested.

This main mechanical challenge category shows us that the morphology of snake teeth can be divided into two groups. Slippery, bulky, and species holding their prey have similar teeth, and their distribution largely overlaps in the morphospace, while species feeding on hard and long prey are similar and overlap, but they barely overlap with the other categories (Figure 4). There are only two species that do not fit in their group: *Atractaspis engaddensis* and *Atractus flammigerus*. *Atractaspis engaddensis* falls within the durophagous group whereas it mostly feeds on small mammals (it would be classified as a bulky eater). Atractaspids are specialized fossorial snakes, they have developed a highly specialized envenomation strategy, which consists of a highly mobile maxilla that can laterally protrude the fang, while the mouth remains closed, allowing the snake to stab its prey backwards. Its dentigerous bones are almost toothless and the hyperspecialization of the envenomation system is associated with a loss of prey manipulation and transport efficiency (Deufel & Cundall, 2003a). A high rate of prey loss in durophagous snakes also having short and stout teeth has been demonstrated (Gripshover & Jayne, 2021), suggesting that this tooth shape may not have evolved to manipulate the prey but rather to prevent breakage. *Atractus flammigerus*, on the other hand, is an earthworm specialist classified in the “long prey” group but falls into the “slippery” category in the morphospace (Figure 4). We strictly defined our main mechanical challenges a priori and classified elongated prey as more constraining than slippery prey, but *Atractus* teeth look more like slippery prey feeders.

### 4.5 Tooth morphology and biomechanical implications

Overall, our results highlight two morphotypes: long, thin, highly curved teeth with a thinner layer of hard tissue (long pulp cavity running along the entire length of the tooth) versus short, stout, straighter with a thicker layer of hard tissue (short pulp cavity). Long, slender teeth are associated with feeding factors that require a good grip on the prey, whether the prey is slippery, or soft, or if there is no solid substrate to support and facilitate feeding. Thin teeth undergo high stresses during penetration into the prey due to bending and are more susceptible to failure (Bar‐On, 2019). Thin teeth are also highly affected by even the slightest axial force that causes high stress (Bar‐On, 2019; Rajabizadeh et al., 2020). Thus, a certain amount of bending might be beneficial to avoid breakage, especially in a feeding context where the prey cannot be correctly restrained. Short and stout teeth are associated with hard or long prey, which both impose either high and/or repeated loading on the teeth, but the stout shape allows to a decrease in the peak stress that originates from the compression force and concentrates it in the tip (Bar‐On, 2019; Rajabizadeh et al., 2020). Rajabizadeh et al. (2020) compared the mechanical properties associated with tooth shape in two sister species; one that eats hard prey and has short, stout teeth versus a generalist with more slender teeth. They used finite element analyses to compare von Mises stress and deformation during loading from various angles on the two teeth. They demonstrated that, as suggested by Bar‐On's results (Bar‐On, 2019), snake teeth barely undergo any stress when applying a tangential force to the tip. Deviation of the applied force from the tangent of the tip imposes a higher and more widely distributed stress in the slender tooth than in the stout one. These results suggest that some of the morphological variations we highlight here may be related to mechanical adaptations of the teeth to dietary constraints. Yet, the two compared shapes are far from representing the large variability in tooth morphology in snakes and many functional aspects of snake teeth remain unexplored such as the effect of curvature or the effect of variation in the inner shape of the teeth on its biomechanical properties.

Conclusions based on the teeth of other vertebrates are hardly applicable to snakes. Snake teeth fulfill different functions than those of other vertebrates; they play a major role in prey capture and intraoral transport, but they are rarely used to reduce the size of prey items. Although snakes have acquired constraining diets such as durophagy, the function of their teeth is not to crush (except for *Fordonia*) but to transport the whole prey into the digestive tract. Therefore, the trade‐off highlighted for the teeth of durophagous vertebrates, between convex teeth that reduce the force needed to break a hard item but increases the strain in the tooth versus concave tooth that reduces the strain but requires higher forces to break the prey, does not apply to durophagous snakes (Crofts, 2015). Our study shows that prey mechanical properties are not the only drivers of tooth morphology, but feeding behavior, and more globally feeding ecology, imposes a variety of constraints that impact their size and shape. Future investigations of the biomechanics of snake teeth may help establish the link between their morphological and behavioral variability and would enrich our understanding of tooth evolution and function in vertebrates. Experimental designs (Kundanati et al., 2020), simulations (Bar‐On, 2019; Rajabizadeh et al., 2020), and analytical tools (Huie et al., 2022) have recently been developed and can be used to better understand the dental biomechanics of snakes using the shapes highlighted in the present study, in a functionally relevant context.

## 5 CONCLUSION

The relationship between dietary ecology and dental morphology has been quite well established in many vertebrate taxa but snakes (Berkovitz & Shellis, 2017). Snakes have a highly specialized feeding behavior and apparatus, which may have constrained the evolution of their tooth morphology and function. Here, we demonstrate that this is not the case; the shape, length, and curvature of snake teeth are highly variable, and this diversity is associated with both prey properties and feeding ecology. Two main tooth shapes were highlighted in our study: short and robust and long and slender teeth. Long teeth are present in snakes that need a good grip on their prey, such as soft‐bodied or bulky prey or snakes feeding underwater. Short teeth are associated with hard and/or long prey items that usually do not involve penetration of the prey. This is the first study quantifying and comparing the morphology of snake teeth in a phylogenetically large sample of species with different ecologies. We hope this will open the way to further investigations on the underlying mechanical properties of snake teeth, thereby improving our understanding of tooth evolution and biomechanics in vertebrates. Finally, we would like to acknowledge the limitation of this study, which is focused on a single tooth per species and one specimen per species. Snakes are considered homodont, yet a quick look at some snakes' dentition is enough to understand that it is not always the case. Some species show a large variation in size, if not shape, of their tooth, even along a single bone (Ryerson & Van Valkenburgh, 2021). From our experience with snakes' dentition, the potential heterodonty may be associated with ecology and especially arboreality. However, it seems that durophagous snakes have similarly sized and shaped teeth. Snakes' dentition has been largely overlooked, and there are a lot of gaps in knowledge to fill.

## AUTHOR CONTRIBUTIONS

**Marion Segall:** Conceptualization (lead); data curation (lead); formal analysis (lead); funding acquisition (lead); investigation (lead); methodology (lead); project administration (lead); visualization (lead); writing – original draft (lead); writing – review and editing (lead). **Celine Houssin:** Data curation (supporting); methodology (supporting); validation (equal); writing – original draft (supporting); writing – review and editing (supporting). **Arnaud Delapré:** Data curation (supporting); methodology (supporting); validation (equal); writing – original draft (supporting); writing – review and editing (supporting). **Raphael Cornette:** Formal analysis (supporting); investigation (supporting); validation (equal); writing – original draft (supporting); writing – review and editing (supporting). **Anthony Herrel:** Data curation (supporting); funding acquisition (supporting); investigation (supporting); validation (equal); writing – original draft (supporting); writing – review and editing (supporting). **Joshua Milgram:** Validation (supporting); writing – original draft (supporting); writing – review and editing (supporting). **Ron Shahar:** Validation (supporting); writing – original draft (supporting); writing – review and editing (supporting). **Maitena Dumont:** Conceptualization (equal); funding acquisition (lead); project administration (lead); validation (equal); writing – original draft (supporting); writing – review and editing (supporting).

## CONFLICT OF INTEREST STATEMENT

The authors declare no conflict of interest.

### OPEN RESEARCH BADGES

This article has earned Open Data and Open Materials badges. Data and materials are available at https://doi.org/10.5061/dryad.wstqjq2rp.

## Supporting information


Data S1.

## Data Availability

The meshes, landmark files, Rdata, Rcode, and all files needed to reproduce this study can be downloaded from the following link https://doi.org/10.5061/dryad.wstqjq2rp
