# Supplementary material for: Armed to the teeth: The underestimated diversity in tooth shape in snakes and its relation to feeding behavior and diet
Source: Ecol Evol. 2023 Apr 13;13(4):e10011. doi: 10.1002/ece3.10011 (PMC10099486; doi:10.1002/ece3.10011)
Supplement: Supplementary file 1 — Data S1. [file ECE3-13-e10011-s001.docx]

**Supplementary Material 1:** List of specimens used in this study along with information about the collection, scans, feeding ecology and teeth measurements. + indicates the major prey item. Teeth length= L_C_ (curvature length), mean angle = D_Cmean_ (average degree of curvature), max angle = D_Cmax_ (maximal degree of curvature).

| **Species** | **Collection** | **Diet** | **Main challenge** | **Shape** | **Hardness** | **Foraging substrate** | **Scan resolution (μm)** | **Tooth length (mm)** | **Mean angle (º)** | **Max angle (º)** | **References** [1] |
| --- | --- | --- | --- | --- | --- | --- | --- | --- | --- | --- | --- |
| *Anilius scytale* | A. Herrel | amphisbaenians (+), caecilians, snakes | long | long | hard | ground | 1.87 | 1.926 | 50.6 | 244.1 | [2,3] |
| *Calabaria reinhardtii* | A. Herrel | rodents | bulky | bulky | soft | ground | 1.42 | 1.278 | 73.4 | 194.0 | [4] |
| *Candoia carinata* | A. Herrel | skinks (+), geckos, frogs, mammals | hold | long | hard | branch | 2.62 | 1.800 | 67.2 | 169.3 | [5] |
| *Eryx jaculus* | HUJI 3634 | mammals | bulky | bulky | soft | ground | 2.00 | 1.847 | 84.2 | 226.5 | [6,7] |
| *Boa constrictor* | Yoan Eynac | mammals, birds | hold | bulky | soft | branch | 7.50 | 6.678 | 92.7 | 291.4 | [8] |
| *Corallus annulatus* | A. Herrel | mammals, birds | hold | bulky | soft | branch | 2.75 | 1.824 | 66.1 | 170.6 | [8,9] |
| *Cylindrophis ruffus* | Ludovic Faure | caecilians (+), snakes, eels | long | long | hard | ground | 1.75 | 1.369 | 67.0 | 143.0 | [10–15] |
| *Python regius* | A. Herrel | mammals, birds | bulky | bulky | soft | ground | 2.15 | 2.288 | 49.3 | 160.2 | [16] |
| *Morelia spilota* | A. Herrel | generalist but mainly mammals (+) | bulky | bulky | soft | ground | 3.37 | 3.813 | 72.0 | 201.5 | [17] |
| *Acrochordus javanicus* | A. Herrel | eleotrid fish | slippery | long | medium | water | 2.27 | 2.628 | 71.8 | 166.9 | [18] |
| *Acrochordus granulatus* | A. Herrel | eleotrid fish, goby-like fish | slippery | long | medium | water | 1.59 | 1.659 | 71.3 | 238.7 | [18–21] |
| *Xenodermus javanicus* | A. Herrel | frog | bulky | bulky | medium | ground | 1.00 | 0.695 | 75.8 | 269.8 | [22] |
| *Aplopeltura boa* | Karine Falco | snails | slippery | long | soft | branch | 1.47 | 1.248 | 65.9 | 243.9 | [23–25] |
| *Pareas carinatus* | Anthony Herrel | snails | slippery | long | soft | branch | 1.22 | 0.551 | 89.8 | 243.1 | [26–29] |
| *Eristicophis macmahoni* | Latoxan | generalist | hold | na | medium | ground | 2.00 | 1.638 | 73.7 | 250.2 | [30–33] |
| *Daboia russelii* | Venomworld | mammals | bulky | bulky | soft | ground | 2.12 | 2.559 | 79.7 | 270.0 | [30,33,34] |
| *Causus sp.* | Ludovic Faure | amphibians | bulky | bulky | medium | ground | 1.59 | 0.757 | 86.1 | 223.0 | [33,35,36] |
| *Echis leucogaster* | Latoxan | centipedes, scorpions, lizards, mammals | hard | long | hard | ground | 1.75 | 0.593 | 57.1 | 153.5 | [30,33,37,38] |
| *Bitis gabonica* | Latoxan | mammals (+), birds | hold | bulky | soft | ground | 3.77 | 3.156 | 87.1 | 279.1 | [30,33,39] |
| *Tropidolaemus wagleri* | AMNH R50991 | mammals | hold | bulky | soft | branch | 1.64 | 1.966 | 58.5 | 120.6 | [40] |
| *Gloydius halys* | Ludovic Faure | mammals | bulky | bulky | soft | ground | 1.18 | 0.889 | 66.7 | 201.6 | [41] |
| *Bothriechis schlegelii* | Venomworld | generalist | hold | na | medium | branch | 2.57 | 2.575 | 51.6 | 164.0 | [1,42] |
| *Agkistrodon piscivorus* | Venomworld | ectotherm generalist | bulky | bulky | medium | ground | 2.00 | 1.656 | 69.5 | 158.8 | [43–47] |
| *Crotalus adamanteus* | A. Herrel | mammals | bulky | bulky | soft | ground | 4.25 | 3.259 | 68.8 | 154.8 | [48] |
| *Subsessor bocourti* | A. Herrel | fish: Osphronemidae (+), Pangasidae, Siluridae, Synbranchidae | slippery | bulky | medium | water | 2.75 | 3.051 | 81.8 | 331.8 | [10,49] |
| *Cantoria violacea* | LKC ZRC 2.3317 | crustaceans (mostly shrimps) | hard | long | hard | ground | 1.00 | 0.969 | 72.0 | 239.2 | [49,50] |
| *Fordonia leucobalia* | LKC ZRC 2.3294 | hard-shelled crustaceans (crabs, lobster) | hard | bulky | hard | ground | 1.59 | 0.827 | 57.3 | 108.5 | [50–52] |
| *Gerarda prevostiana* | LKC ZRC 2.3292 | crabs | hard | bulky | hard | ground | 1.00 | 0.625 | 64.1 | 153.4 | [49–51] |
| *Homalopsis buccata* | A. Herrel | small or long fish (*Lebistes reticulatus, Mystus, Monopterus albus, Clarias sp, Puntius binotatus*) | slippery | long | medium | water | 1.70 | 2.263 | 87.1 | 295.9 | [10,49,53] |
| *Malpolon insignitus* | HUJI 16560 | generalist (mostly mammals) | bulky | bulky | soft | ground | 1.81 | 1.570 | 57.3 | 130.5 | [54–56] |
| *Atractaspis engaddensis* | HUJI 16567 | mammals (+), lizards, snakes | bulky | bulky | soft | ground | 1.75 | 0.430 | 60.1 | 181.8 | [57] |
| *Micrurus psyches* | A. Herrel | snakes | long | long | hard | ground | 0.97 | 0.545 | 68.9 | 112.8 | [58] |
| *Ophiophagus hannah* | Latoxan | snakes | long | long | hard | ground | 2.27 | 1.203 | 64.0 | 149.7 | [59,60] |
| *Dendroaspis viridis* | Latoxan | mammals, birds | hold | bulky | soft | branch | 1.50 | 0.987 | 71.4 | 281.2 | [61] |
| *Naja annulata* | Latoxan | fish | slippery | long | medium | water | 2.50 | 2.682 | 80.3 | 211.4 | [62] |
| *Naja nigricollis* | Latoxan | generalist | bulky | na | medium | ground | 1.50 | 1.199 | 62.4 | 132.7 | [63] |
| *Laticauda colubrina* | AMNH R38111 | eels | long | long | medium | water | 1.06 | 0.819 | 112.9 | 77.3 | [52,64–69] |
| *Aipysurus laevis* | Latoxan | small or long fish (catfish, small snappers) | slippery | long | medium | water | 2.37 | 2.244 | 90.8 | 200.0 | [52,66,67,70,71] |
| *Hydrophis platurus* | A. Herrel | small fish | slippery | long | medium | water | 1.92 | 2.347 | 99.6 | 280.2 | [52,70,72–79] |
| *Grayia ornata* | A. Herrel | long fish (siluriform) | slippery | long | medium | branch | 1.29 | 1.559 | 91.5 | 276.7 | [62,80] |
| *Boiga dendrophila* | Latoxan | mammals, birds | hold | bulky | soft | branch | 1.83 | 2.134 | 63.9 | 167.1 | [81] |
| *Boiga cynodon* | Latoxan | generalist | hold | na | medium | branch | 2.00 | 2.034 | 73.4 | 129.9 | [81] |
| *Dasypeltis scabra* | A. Herrel | eggs | hard | bulky | hard | ground | 1.04 | 0.572 | 67.5 | 248.8 | [82] |
| *Dispholidus typus* | Latoxan | birds, chameleons, agama, toad | hold | bulky | medium | branch | 1.75 | 1.062 | 57.7 | 154.1 | [83] |
| *Philothamnus semivariegatus* | Latoxan | lizards (gecko) | hold | bulky | medium | branch | 2.00 | 0.736 | 74.0 | 176.0 | [61,80,84] |
| *Eirenis decemlineatus* | HUJI 4780 | arthropods (orthopterans, insects, spiders, scorpions, centipedes) | hard | bulky | hard | ground | 1.50 | 0.495 | 52.6 | 98.9 | [85,86] |
| *Eirenis lineomaculatus* | HUJI 16485 | arthropods (orthopterans, centipedes, spiders, scorpions) | hard | bulky | hard | ground | 1.75 | 0.475 | 75.6 | 158.1 | [85,86] |
| *Oxybelis aeneus* | A. Herrel | generalist (mostly elongated lizards) | hold | long | medium | branch | 1.12 | 0.998 | 82.0 | 209.2 | [87,88] |
| *Scolecophis atrocinctus* | A. Herrel | centipedes | hard | long | hard | ground | 1.01 | 0.368 | 64.6 | 181.8 | [89] |
| *Coluber constrictor* | AMNH R27367 | generalist (snakes (+), elongated lizards…) | long | long | hard | ground | 1.25 | 1.449 | 64.0 | 157.1 | [90–92] |
| *Stenorrhina degenhardtii* | AMNH R38087 | arthropods (mostly spiders) | hard | bulky | hard | ground | 1.59 | 0.567 | 56.2 | 128.0 | [93–95] |
| *Gonyosoma boulengeri* | A. Herrel | endotherm generalist | hold | bulky | soft | branch | 1.50 | 0.776 | 83.1 | 284.0 | [96] |
| *Coronella girondica* | A. Herrel | elongated lizards (skinks, lacertids, anguis) | long | long | hard | ground | 1.40 | 0.786 | 63.8 | 166.7 | [97] |
| *Lampropeltis triangulum* | AMNH R177123 | elongated lizards (+, skinks, *Sceloporus*), snakes, mammals | long | long | medium | ground | 1.22 | 0.668 | 46.3 | 165.7 | [98] |
| *Natrix tessellata* | HUJI 16537 | mostly elongated fish (goby, Cyprinidae…) | slippery | long | medium | water | 1.50 | 1.165 | 74.5 | 228.5 | [99–101] |
| *Liodytes rigida* | AMNH R177031 | crayfish | hard | bulky | hard | ground | 1.00 | 0.672 | 49.9 | 147.2 | [102–105] |
| *Heterodon nasicus* | Yoan Eynac | reptiles, toads, eggs, small turtles | bulky | bulky | hard | ground | 1.30 | 1.255 | 82.3 | 267.4 | [106–109] |
| *Imantodes cenchoa* | Vincent Prémel | elongated thin scaled lizards (mostly anoles, +, *Gonatodes* *sp.*), amphibians | hold | long | medium | branch | 1.00 | 0.521 | 87.3 | 299.3 | [3,110] |
| *Atractus flammigerus* | MNHN 80-24 | earthworms | long | long | soft | ground | 1.25 | 1.107 | 70.8 | 193.7 | [3,111,112] |
| *Sibon sp.* | A. Herrel | amphibian eggs, earthworms, slugs, | slippery | long | soft | branch | 1.02 | 1.058 | 83.9 | 236.1 | [113,114] |
| *Helicops sp.* | A. Herrel | mostly fish (Perciformes, Characiformes, Cyprinodontiformes…) | slippery | long | medium | water | 1.20 | 1.252 | 70.6 | 248.5 | [3,115–117] |
| *Siphlophis compressus* | Vincent Prémel | mostly elongated thin-scales lizards (anoles, *Gonatodes*) | long | long | medium | ground | 1.59 | 0.663 | 62.5 | 192.4 | [1,3] |
| *Clelia clelia* | MNHN 79-47 | reptiles (mostly snakes +) | long | long | hard | ground | 1.40 | 0.529 | 52.0 | 189.5 | [1] |

**References**

1. Grundler MC. 2020 SquamataBase: A natural history database and R package for comparative biology of snake feeding habits. *Biodivers. Data J.* **8**. (doi:10.3897/BDJ.8.E49943)

2. Maschio GF, da Prudente ALC, da Rodrigues FS, Hoogmoed MS. 2010 Food habits of *Anilius scytale* (Serpentes: Aniliidae) in the Brazilian Amazonia. *Zoologia* **27**, 184–190. (doi:10.1590/S1984-46702010000200005)

3. Martins M, Oliveira ME. 1998 Natural history of snakes in forests of the Manaus Region, Central Amazonia, Brazil. *Herpetol. Nat. Hist.* **6**, 85–150.

4. Luiselli L, Effah C, Angelici FM, Odegbune E, Inyang MA, Akani GC, Politano E. 2002 Female breeding frequency, clutch size and dietary habits of a Nigerian population of Calabar ground python, Calabaria reinhardtii. *Herpetol. J.* **12**, 127–129.

5. Harlow P, Shine R. 1992 Food habits and reproductive biology of the Pacific Island boas (Candoia). *J. Herpetol.* **26**, 60–66.

6. Faraone FP, Barra SA, Giacalone G, Chiara R, Russotto S, Lo Valvo M. 2017 First observations of oophagy in a wild population of the sand boa (Eryx jaculus). *Herpetol. Bull.* **142**, 48–49.

7. Faraone FP, Russotto S, Giacalone G, Valvo M Lo, Belardi I, Mori E. 2021 Food habits of the Javelin Sand Boa *Eryx jaculus* (Linnaeus 1758; Serpentes, Erycidae) in Sicily, Italy. *J. Herpetol.* **55**, 452–458. (doi:10.1670/20-047)

8. Pizzatto L, Marques OA V., Facure K. 2010 Food habits of Brazilian boid snakes: Overview and new data, with special reference to *Corallus hortulanus*. *Amphib. Reptil.* **30**, 533–544. (doi:10.1163/156853809789647121)

9. Henderson RW, Pauers MJ. 2012 On the diets of Neotropical Treeboas (Squamata: Boidae: *Corallus*). *South Am. J. Herpetol.* **7**, 172–180. (doi:10.2994/057.007.0207)

10. Brooks SE, Allison EH, Gill JA, Reynolds JD. 2009 Reproductive and trophic ecology of an assemblage of aquatic and semi-aquatic snakes in Tonle Sap, Cambodia. *Copeia* **2009**, 7–20. (doi:10.1643/CE-07-102)

11. Kupfer A, Gower DJ, Himstedt W. 2003 Field observations on the predation of the caecilian amphibian, genus *Ichthyophis* (Fitzinger,1826), by the red-tailed pipe snake Cylindrophis ruffus (Laurenti, 1768). *Amphibia-Reptilia* **24**, 212–215. (doi:10.1163/156853803322390462)

12. Greene HW. 1983 Dietary correlates of the origin and radiation of snakes. *Am. Zool.* **23**, 431–441.

13. Smith MA. 1943 The Fauna of British India, Ceylon and Burma, including the whole of the Indo-Chinese sub-region. In *Reptilia and Amphibia*, p. 606. London.

14. Cundall D. 1995 Feeding behaviour in *Cylindrophis* and its bearing on the evolution of alethinophidian snakes. *J. Zool.* **237**, 353–376. (doi:10.1111/j.1469-7998.1995.tb02767.x)

15. Da Silva MAO, Heegaard S, Wang T, Gade JT, Damsgaard C, Bertelsen MF. 2017 Morphology of the snake spectacle reflects its evolutionary adaptation and development. *BMC Vet. Res.* **13**, 1–8. (doi:10.1186/s12917-017-1193-2)

16. Luiselli L, Angelici FM. 1998 Sexual size dimorphism and natural history traits are correlated with intersexual dietary divergence in royal pythons (Python regius) from the rainforests of southeastern Nigeria. *Ital. J. Zool.* **65**, 183–185. (doi:10.1080/11250009809386744)

17. Shine R. 1991 Why do larger snakes eat larger prey items? *Funct. Ecol.* **5**, 493–502.

18. Lillywhite HB. 2003 File snakes (Acrochordidae). In *Grzimek’s Animal Life Encyclopedia, 2nd Edition, Volume 7, Reptiles.* (eds M Hutchins, JB Murphy, N Schlager), pp. 439–444. Farmington Hills, MI: Gale Group.

19. Garcia VOS, Papa RDS, Briones JCA, Mendoza N, Okuda N, Diesmos AC. 2014 Food habits and distribution of the Lake Taal Sea Snake (*Hydrophis semperi Garman* 1881) and the sympatric little file snake (*Acrochordus granulatus* Schneider 1799) in Lake Taal, Philippines. *Asian Herpetol. Res.* **5**, 255–262. (doi:10.3724/SP.J.1245.2014.00255)

20. Voris HK, Glodek GS. 1980 Habitat, diet, and reproduction of the file snake, *Acrochordus granulatus*, in the straits of Malacca. *J. Herpetol.* **14**, 105–108.

21. Shine R, Houston D. 1993 Family Acrochordidae. In *Fauna of Australia*, pp. 322–324.

22. Vallejos JG, Ahmad Sah HH. 2017 Limnonectes palavanensis (Smooth Guardian Frog). Predation. *Herpetol. Rev.* **48**, 608–609.

23. Hoso M, Asami T, Hori M. 2007 Right-handed snakes: convergent evolution of asymmetry for functional specialization. *Biol. Lett.* **3**, 169–172. (doi:10.1098/rsbl.2006.0600)

24. Kojima Y, Fukuyama I, Kurita T, Hossman MY Bin, Nishikawa K. 2020 Mandibular sawing in a snail-eating snake. *Sci. Rep.* **10**, 1–6. (doi:10.1038/s41598-020-69436-7)

25. Stuebing RB, Inger RF. 1999 *A field guide to the snakes of Borneo*. Sabah, Malaysia: National History Publications (Borneo).

26. Hoso M. 2017 Asymmetry of mandibular dentition is associated with dietary specialization in snail-eating snakes. *PeerJ* **5**, e3011. (doi:10.7717/peerj.3011)

27. Danaisawadi P, Asami T, Ota H, Sutcharit C, Panha S. 2016 A snail-eating snake recognizes prey handedness. *Sci. Rep.* **6**, 1–8. (doi:10.1038/srep23832)

28. Danaisawadi P, Asami T, Ota H, Sutcharit C, Panha S. 2016 Predatory behavior of the snail-eating snake *Pareas carinatus* (Boie, 1828) (Squamata: Pareidae): an ethogram study. *Trop. Nat. Hist.* **16**, 21–31.

29. Cundall D, Greene HW. 2000 Feeding in Snakes. In *Feeding: Form, Function and Evolution in Tetrapod Vertebrates* (ed K Schwenk), pp. 293–333. London, United Kingdom: Academic Press. (doi:10.1016/b978-012632590-4/50010-1)

30. Mallow D, Ludwig D, Nilson G. 2003 *True Vipers: Natural History and Toxinology of Old World Vipers*. Malabar, FL, USA: Kreiger Publishing Company.

31. op den Brouw B *et al.* 2021 Extensive variation in the activities of *Pseudocerastes* and *Eristicophis* viper venoms suggests divergent envenoming strategies are used for prey capture. *Toxins (Basel).* **13**. (doi:10.3390/toxins13020112)

32. Minton SA. 1966 A contribution to the herpetology of West Pakistan. *Bull. Am. Museum Nat. Hist.* **134**, 27-184 ST-A contribution to the herpetology of.

33. Phelps T. 2010 *Old World Vipers, A Natural History of the Azemiopinae and Viperinae*. Frankfurt am Main, Germany: Edition Chimaira.

34. Wüster W. 1998 The genus *Daboia* (Serpentes: Viperidae): Russell’s viper. *Hamadryad*. **23**, 33–40.

35. Akani GC, Luiselli L, Tooze Z, Angelici FM, Corti C, Zuffi MAL. 2001 The ecological distribution of *Causus* Wagler 1830 (Viperidae) in Nigeria, with special reference to *C. resimus* (Peters 1862) and *C. lichtensteini* (Jan 1859), two species rarely recorded from this country. *Trop. Zool.* **14**, 185–195. (doi:10.1080/03946975.2001.10531151)

36. Spawls S, Branch B. 1995 *The dangerous snakes of Africa*. London: Blandford.

37. Barlow A, Pook CE, Harrison RA, Wüster W. 2009 Coevolution of diet and prey-specific venom activity supports the role of selection in snake venom evolution. *Proc. R. Soc. B Biol. Sci.* **276**, 2443–2449. (doi:10.1098/rspb.2009.0048)

38. Escoriza D, Metallinou M, Donaire-Barroso D, Amat F, Carranza S. 2010 Biogeography of the White-Bellied Carpet viper *Echis leucogaster* Roman, 1972 in Morocco, a study combining mitochondrial DNA data and ecological niche modeling. *Butll. la Soc. Catalana d’Herpetologia* **18**, 55–68.

39. Luiselli L, Akani GC. 2003 Diet of sympatric gaboon vipers (*Bitis gabonica*) and nose-horned vipers (*Bitis nasicornis*) in Southern Nigeria. *African J. Herpetol.* **52**, 101–106. (doi:10.1080/21564574.2003.9635485)

40. Orlov Ni, Ananjeva NB, Khalikov R. 2000 Natural History of Pitvipers in Eastern and Southeastern Asia. *Biol. Vipers* , 345–360.

41. Simonov E. 2009 Differences in habitat use, daily activity patterns and preferred ambient temperatures of adult and neonate *Gloydius halys halys* from an isolated population in southwest Siberia: Preliminary data. *Herpetol. Notes* **2**, 1–7.

42. Sorrell GG. 2009 Diel movement and predation activity patterns of the eyelash Palm-pitviper (*Bothriechis schlegelii*). *Copeia* , 105–109. (doi:10.1643/CE-06-284)

43. McKnight DT, Harmon JR, McKnight JL, Ligon DB. 2014 Notes on the diets of seven sympatric snakes in the genera Agkistrodon, Nerodia, Sistrurus, and Thamnophis. *Herpetol. Notes* **7**, 171–177.

44. Vincent SE, Herrel A, Irschick DJ. 2004 Sexual dimorphism in head shape and diet in the cottonmouth snake (*Agkistrodon piscivorus*). *J. Zool.* **264**, 53–59. (doi:10.1017/S0952836904005503)

45. Lillywhite HB, McCleary RJR. 2008 Trophic Ecology of Insular Cottonmouth Snakes: Review and Perspective. *South Am. J. Herpetol.* **3**, 175–185. (doi:10.2994/1808-9798(2008)3[175:TEOICS]2.0.CO;2)

46. Burtkett RD. 1966 Natural history of the cottonmouth moccasin, *Agkistrodon piscivorus* (Reptilia). *Univ. Kansas Publ. Museum Nat. Hist.* **17**, 435–491.

47. Young BA, Aguiar A, Lillywhite HB. 2008 Foraging cues used by insular Florida cottonmouths, *Agkistrodon piscivorus conanti*. *South Am. J. Herpetol.* **3**, 135–144. (doi:10.2994/1808-9798(2008)3[135:fcubif]2.0.co;2)

48. Timmerman WW. 1995 Home range, habitat use, and behavior of the eastern diamondback rattlesnake (*Crotalus adamanteus*) on the Ordway Preserve. *Bull. Florida Museum Nat. Hist.* **38**, 127–158.

49. Murphy JC. 2007 *Homalopsid snakes: evolution in the mud*. Malabar, Florida: Krieger Publishing Company.

50. Voris HK, Murphy JC. 2002 The prey and predators of Homalopsine snakes. *J. Nat. Hist.* **36**, 1621–1632. (doi:10.1080/00222930110062642)

51. Jayne BC, Voris HK, Ng PKL. 2018 How big is too big? Using crustacean-eating snakes (Homalopsidae) to test how anatomy and behaviour affect prey size and feeding performance. *Biol. J. Linn. Soc.* **123**, 636–650. (doi:10.1093/biolinnean/bly007)

52. Heatwole H. 1999 *Sea snakes*. Kensington, NSW, Australia, Australia: University of New South Wales Press Ltd.

53. Berry PY, Lim GS. 1967 The breeding pattern of the puff-faced water snake, *Homalopsis buccata* Boulenger. *Copeia* **1967**, 307–313.

54. Cattaneo A. 2015 Contributo alla conoscenza dei serpenti delle isole del Canale di Sicilia. *Nat. Sicil.* **XXXIX**, 3–28.

55. Safaei-Mahroo B, Ghaffari H, Salmabadi S, Kamangar A, Almasi S, Kazemi SM, Ghafoor A. 2017 Eastern Montpellier snake (*Malpolon insignitus fuscus*) Ophiophagy behavior from Zagros mountains. *Russ. J. Herpetol.* **24**, 69–72. (doi:10.30906/1026-2296-2019-24-1-69-72)

56. Plettenberg-Laing A. 2021 Eyes bigger than the stomach? A record of the eastern Montpellier snake, *Malpolon insignitus* (Geoffroy Saint-Hilaire, 1827), preying on an adult glass lizard, *Pseudopus apodus* (Pallas, 1775). *Herpetol. Notes* **14**, 361–363.

57. Al-Sadoon MK, Paray BA, Rudayni HA, Al-Mfarij AR, Albeshr MF. 2020 Seasonal food composition of a burrowing asp, *Atractaspis engaddensis* Haas, 1950 from natural habitats of an arid Arabian desert. *J. King Saud Univ. - Sci.* **32**, 2393–2396. (doi:10.1016/j.jksus.2020.03.022)

58. Beebe W. 1946 Field notes on the snakes of Kartabo, British Guiana, and Caripito, Venezuela. *Zool. Sci. Contrib. New York Zool. Soc.* **31**, 11–52. (doi:10.5962/p.203521)

59. Jones MD, Crane MS, Silva IMS, Artchawakom T, Waengsothorn S, Suwanwaree P, Strine CT, Goode M. 2020 Supposed snake specialist consumes monitor lizards: diet and trophic implications of king cobra feeding ecology. *Ecology* **101**, 1–4. (doi:10.1002/ecy.3085)

60. Bhaisare D, Ramanuj V, Shankar PG, Vittala M, Goode M, Whitaker R, El-Dairi M, House RJ. 2010 Observations on a wild King Cobra (*Ophiophagus hannah*), with emphasis on foraging and diet. *IRCF Reptil. Amphib.* **17**, 95–102. (doi:10.1016/B978-0-323-60984-5.00062-7)

61. Trape J-F, Mané Y. 2006 *Guide des serpents d’Afrique occidentale*. Marseille, France: IRD Éditions. (doi:10.4000/books.irdeditions.37282)

62. Chippaux J-P. 2006 *Les serpents d’Afrique occidentale et centrale*.

63. Luiselli L, Angelici FM, Akani GC. 2002 Comparative feeding strategies and dietary plasticity of the sympatric cobras *Naja melanoleuca* and *Naja nigricollis* in three diverging Afrotropical habitats. *Can. J. Zool.* **80**, 55–63. (doi:10.1139/z01-178)

64. Shine R, Reed RR, Shetty S, Cogger HG. 2002 Relationships between sexual dimorphism and niche partitioning within a clade of sea-snakes (Laticaudinae). *Oecologia* **133**, 45–53. (doi:10.1007/s00442-002-1012-7)

65. Voris HK. 1972 The role of sea snakes (Hydrophiidae) in the trophic structure of coastal ocean communities. *J. Mar. Biol. Assoc. India* **14**, 429–442.

66. McCosker JE. 1975 Feeding behavior of Indo-Australian Hydrophiidae. In *The biology of sea snakes* (ed WA Dunson), pp. 217–232. Baltimore, Maryland: University Park Press.

67. Ineich I, Laboute P. 2002 *Sea snakes of New Caledonia*. IRD Editio. Paris.

68. Pernetta JC. 1977 Observations on the habits and morphology of the sea snake *Laticauda colubrina* (Schneider) in Fiji. *Can. J. Zool.* **55**, 1612–1619. (doi:10.1139/z77-210)

69. Shetty S, Shine R. 2002 Sexual divergence in diets and morphology in Fijian sea snakes Laticauda colubrina (Laticaudinae). *Austral Ecol.* **27**, 77–84. (doi:10.1046/j.1442-9993.2002.01161.x)

70. Sherratt E, Rasmussen AR, Sanders KL. 2018 Trophic specialization drives morphological evolution in sea snakes. *R. Soc. Open Sci.* **5**, 1–8. (doi:10.1098/rsos.172141)

71. Limpus CJ. 1975 Coastal sea snakes of subtropical Queensland Waters (23° to 28° South Latitude). In *The biology of sea snakes* (ed WA Dunson), pp. 173–182. Baltimore, Maryland: University Park Press.

72. Voris HK, Voris HH. 1983 Feeding Strategies in Marine Snakes: An Analysis of Evolutionary, Morphological, Behavioral and Ecological Relationships. *Am. Zool.* **23**, 411–425.

73. Bessesen BL, Galbreath GJ. 2017 A new subspecies of sea snake, *Hydrophis platurus xanthos*, from Golfo Dulce, Costa Rica. *Zookeys* **686**, 109–123. (doi:10.3897/zookeys.686.12682)

74. Dunson WA, Ehlert GW. 1971 Effects of temperature, salinity, and surface water flow on distribution of the sea snake Pelamis. *Limnol. Oceanogr.* **16**, 845–853.

75. Rubinoff I, Graham JB, Motta J. 1986 Diving of the sea snake Pelamis platurus in the Gulf of Panamá - I. Dive depth and duration. *Mar. Biol.* **91**, 181–191. (doi:10.1007/BF00569434)

76. Rubinoff I, Graham JB, Motta J. 1988 Diving of the sea snake *Pelamis platurus* in the Gulf of Panama II. Horizontal movement patterns. *Mar. Biol.* **97**, 157–163. (doi:10.1126/science.333.6043.686)

77. Hecht MK, Kropach CN, Hecht BM. 1974 Distribution of the Yellow-Bellied Sea Snake, *Pelamis platurus*, and its significance in relation to the fossil record. *Herpetologica* **30**, 387–396.

78. Hibbard E, Lavergne J. 1972 Morphology of the retina of the sea-snake, *Pelamis platurus*. *J. Anat.* **112**, 125–136.

79. Ineich I. 1988 Le serpent marin (*Pelamis platurus*) (Elapidae, Hydrophiinae) : Bilan des connaissances sur sa biologie et sa distribution; situation en Polyn̩sie Orientale. *Ann̩ée Biol.* **27**, 93–117.

80. Akinpelu AI, Areo A. 2007 The Snakes of Osun Grove : a world heritage site in Osogbo, Nigeria. *Rev. Biol. Trop.* **55**, 717–721.

81. Greene HW. 1989 Ecological, evolutionary, and conservation implications of feeding biology in Old World cat snakes, genus *Boiga* (Colubridae). *Proc. Calif. Acad. Sci.* **46**, 193–207.

82. Gans C. 1952 The functional morphology of the egg-eating adaptations in the snake genus Dasypeltis. *Zool. New York Zool. Soc.* **37**, 209–244.

83. Smith CCD, Layloo I, Maritz RA, Maritz B. 2019 Sexual dichromatism does not translate into sex-based difference in morphology or diet for the African boomslang. *J. Zool.* **308**, 253–258. (doi:10.1111/jzo.12670)

84. Spawls S, Howell K, Hinkel H, Menegon M. 2018 *Field Guide to East African Reptiles*. Bloomsbury Publishing.

85. Werner YL. 2016 *Reptile Life in the Land of Israel*. Edition Ch.

86. Mahlow K, Tillack F, Schmidtler JF, Müller J. 2013 An annotated checklist, description and key to the dwarf snakes of the genus *Eirenis* Jan, 1863 (Reptilia: Squamata: Colubridae), with special emphasis on the dentition. *Vertebr. Zool.* **63**, 41–85.

87. Hetherington TE. 2006 *Oxybelis aeneus* (brown Vinesnake). Diet. *Herpetol. Rev.* **37**, 94–95.

88. Henderson RW, Binder MH. 1980 The ecology and behavior of Vine Snakes (Ahaetulla, Oxybelis, Thelotornis, Uromacer): A review. *Milwaukee Public Museum Contrib. Biol. Geol.* **37**, 1–39.

89. Jackson TNW, Jouanne H, Vidal N. 2019 Snake Venom in Context: Neglected Clades and Concepts. *Front. Ecol. Evol.* **7**, 1–9. (doi:10.3389/fevo.2019.00332)

90. Gehlbach FR. 2010 Suburbanization of a Central Texas herpetofauna. *IRCF Reptil. Amphib.* **17**, 87–93.

91. Klimstra WD. 1959 Foods of the racer, *Coluber constrictor*, in Southern Illinois. *Copeia* **1959**, 210–214.

92. Fitch HS. 1963 Natural history of the racer *Coluber constrictor*. *Univ. Kansas Publ. Museum Nat. Hist.* **15**, 351–468.

93. Hutchins M, Murphy JB, Schlager N, editors. 2003 *Grzimek’s Animal Life Encyclopedia, 2nd Edition. Volume 7, Reptiles*. Farmington Hills, MI: Gale Group.

94. Sexton OJ, Heatwole H. 1965 Life history notes on some Panamanian snakes. *Caribb. J. Sci.* **5**, 39–43.

95. Duellman WE. 1963 Amphibians and Reptiles of the Rainforests of Southern El Petén, Guatemala. *Univ. Kansas Publ. Museum Nat. Hist.* **15**, 205–249.

96. Hecht VL, Pham CT, Nguyen TT, Nguyen TQ, Bonkowski M, Ziegler T. 2013 First report on the herpetofauna of Tay Yen Tu Nature Reserve, northeastern Vietnam. *Biodivers. J.* **4**, 507–552.

97. Luiselli L, Pleguezuelos JM, Capula M, Villafranca C. 2001 Geographic variation in the diet composition of a secretive mediterranean colubrid snake: *Coronella girondica* from Spain and Italy. *Ital. J. Zool.* **68**, 57–60. (doi:10.1080/11250000109356383)

98. Hamilton BT, Hart R, Sites JW. 2012 Feeding ecology of the milksnake (*Lampropeltis triangulum*, Colubridae) in the Western United States. *J. Herpetol.* **46**, 515–522. (doi:10.1670/10-091)

99. Filippi E, Capula M, Luiselli L, Agrimi U. 1996 The prey spectrum of *Natrix natrix* (Linnaeus, 1758) and *Natrix tessellata* (Laurenti, 1768) in sympatric populations. *Herpetozoa* **8**, 155–164.

100. Luiselli L, Capizzi D, Filippi E, Anibaldi C, Rugiero L, Capula M. 2007 Comparative diets of three populations of an aquatic snake (*Natrix tessellata*, Colubridae) from Mediterranean streams with different hydric regimes. *Copeia* **2007**, 426–435.

101. Göçmen B, Çiçek K, Yildiz MZ, Atatür MK, Dinçaslan YE, Mebert K. 2011 A preliminary study on the feeding biology of the dice snake, *Natrix tessellata*, in Turkey. *Mertensiella* **18**, 365–369.

102. Gibbons JW, Dorcas ME. 2004 *North American watersnakes, a natural history*. Animal Nat. University of Oklahoma Press.

103. Waters RM, Burghardt GM. 2013 Prey availability influences the ontogeny and timing of chemoreception-based prey shifting in the striped crayfish snake, *Regina alleni*. *J. Comp. Psychol.* **127**, 49–55. (doi:10.1037/a0029267)

104. Kofron CP. 1978 Foods and habitats of aquatic snakes (Reptilia, Serpentes) in a Louisiana swamp. *J. Herpetol.* **12**, 543–554.

105. Tumlison R, Roberts KG. 2018 Prey Handling Behavior in the Gulf Crayfish Snake (*Liodytes rigida*). *Herpetol. Conserv. Biol.* **13**, 617–621.

106. Durso AM, Mullin SJ. 2017 Ontogenetic shifts in the diet of plains hog-nosed snakes (*Heterodon nasicus*) revealed by stable isotope analysis. *Zoology* **120**, 83–91. (doi:10.1016/j.zool.2016.07.004)

107. Adams AAY, Martin DJ, Adams RD. 2015 *Heterodon nasicus* (Plains Hog-nosed snake). Diet. *Herpetol. Rev.* **46**, 645.

108. Ernst CH, Ernst EM. 2003 *Snakes of the United States and Canada*. Washington,: Smithsonian Instituton Press.

109. Averill-Murray RC. 2006 Natural history of the western hognose snake (*Heterodon nasicus*) with notes on envenomation. *Son. Herpetol.* **19**, 98–101.

110. de Sousa KRM, Prudente ALC, Maschio GF. 2014 Reproduction and diet of *Imantodes cenchoa* (Dipsadidae: Dipsadinae) from the Brazilian Amazon. *Zoologia* **31**, 8–19. (doi:10.1590/S1984-46702014000100002)

111. Cunha OR, Nascimiento FP. 1993 Ofídios da Amazônia. As cobras da região Leste do Pará. *Bol. do Mus. Para. Hist. Nat. e Ethnogr.* **9**, 1–191.

112. Martins M, Oliveira ME. 1993 The snakes of the genus *Atractus* Wagler (Reptilia: Squamata: Colubridae) from the Manaus region, central Amazonia, Brazil. *Zool. Meded.* **67**, 21–40. (doi:10.11606/issn.2316-9079.v16i2p225-242)

113. Kofron CP. 1988 Systematics of Neotropical gastropod-eating snakes: The *sartorii* group of the genus Sibon. *Amphibia-Reptilia* **9**, 145–168. (doi:10.1163/156853888X00558)

114. Ray JM, Montgomery CE, Mahon HK, Savitzky AH, Lips KR. 2012 Goo-eaters: Diets of the neotropical snakes *Dipsas* and *Sibon* in central panama. *Copeia* , 197–202. (doi:10.1643/CH-10-100)

115. De Carvalho Teixeira C, De Assis Montag LF, Dos Santos-Costa MC. 2017 Diet composition and foraging habitat use by three species of water snakes, *Helicops* Wagler, 1830, (Serpentes: Dipsadidae) in Eastern Brazilian Amazonia. *J. Herpetol.* **51**, 215–222. (doi:10.1670/15-161)

116. Scartozzoni RR. 2009 Estratégias reprodutivas e ecologia alimentar de serpentes aquáticas da tribo Hydropsini (Dipsadidae, Xenodontinae).

117. Strussmann C, Sazima I. 1993 The snake assemblage of the Pantanal at Pocone, Western Brazil: faunal composition and ecological summary. *Stud. Neotrop. Fauna Environ.* **28**, 157–168. (doi:10.1080/01650529309360900)


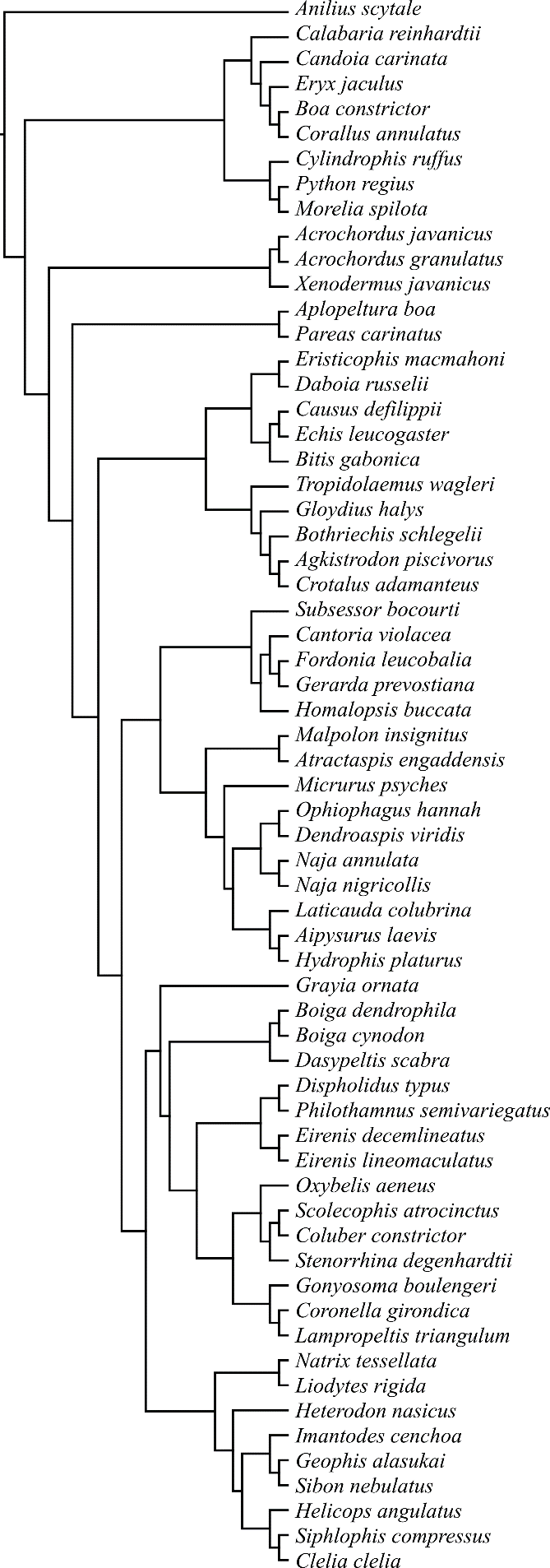
**Supplementary Material 2a:** Phylogeny of the species included in our sample. Tree pruned from the phylogeny presented in Pyron *et al.* 2014. Species were absent from the phylogeny were replaced by their closest relative in the phylogeny: *Malpolon insignitus = Malpolon monspessulanus, Atractaspis engaddensis = Atractaspis bibronii, Scolecophis atrocinctus = Tantilla melanocephala, Stenorrhina degenhardtii = Stenorrhina freminvillei*

**Supplementary Material 2b:** Snapshots of the 3D reconstructions based on the CTscans for each species along with the phylogeny. Frame color corresponds to prey hardness. This figure shows that dentary teeth are rather similar in shape, although they can vary in size for a few species. Most of the intra-individual variation seen in the snapshot comes from teeth that are being replaced or not fully grown and functional.


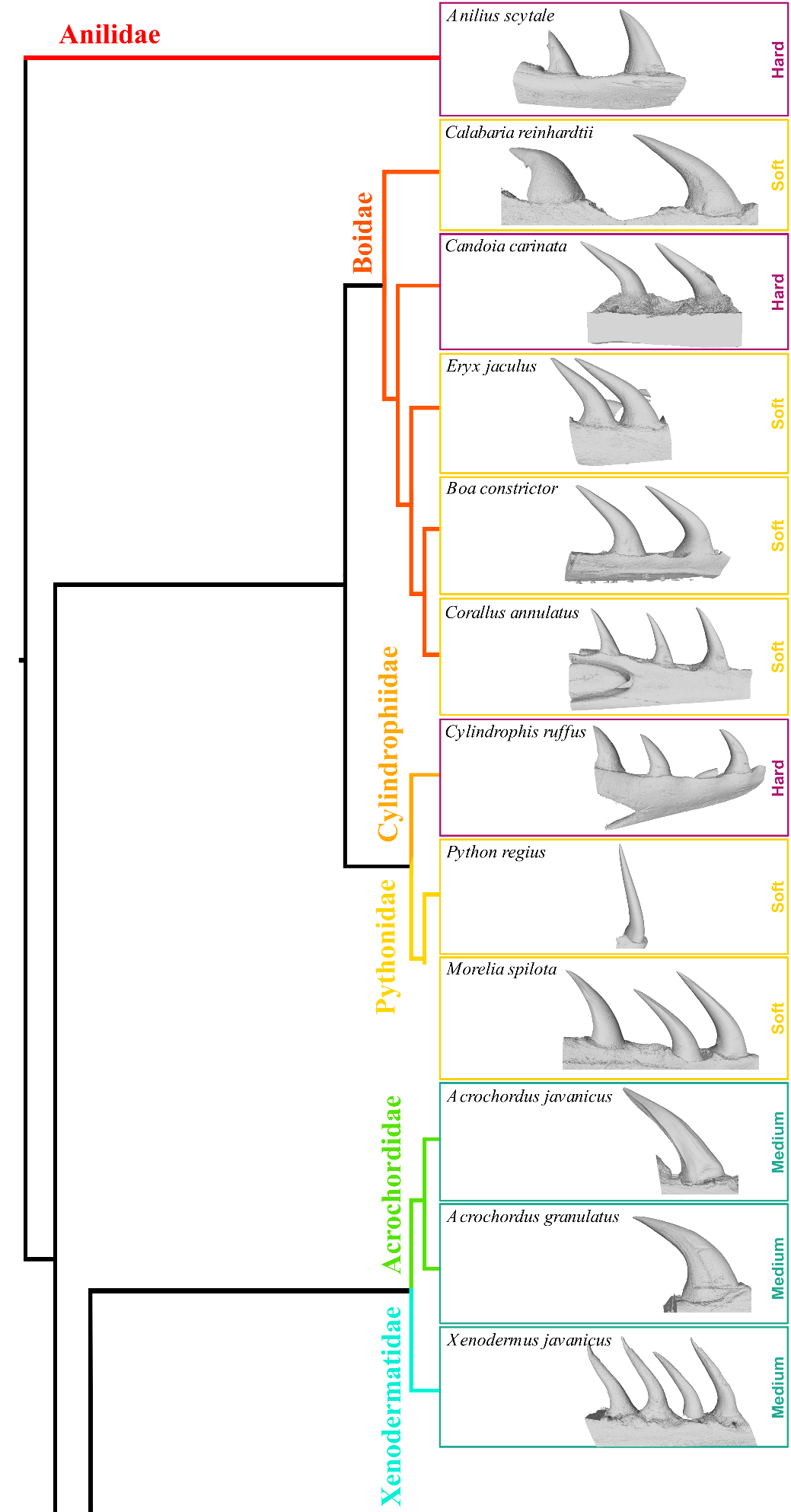


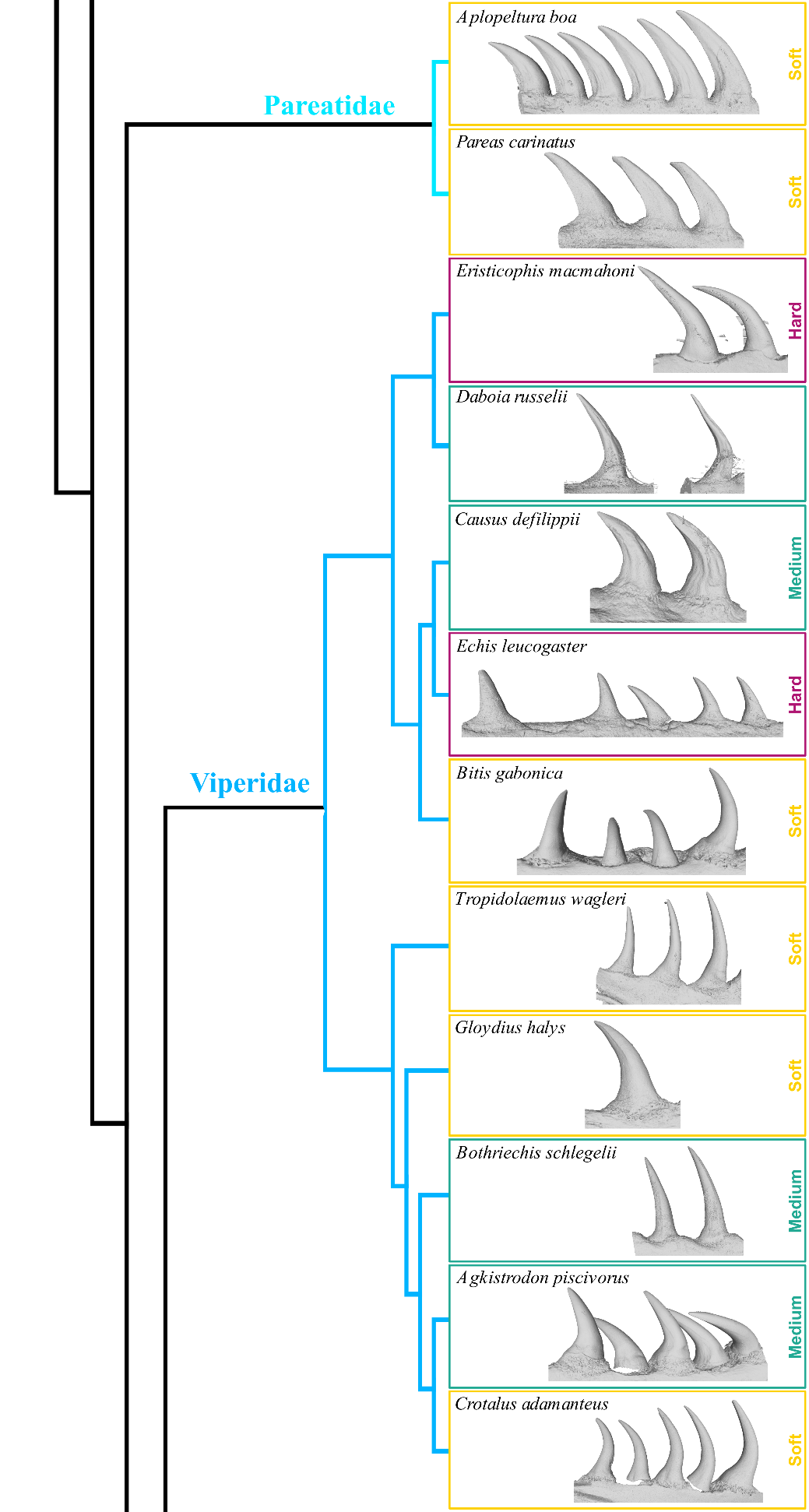


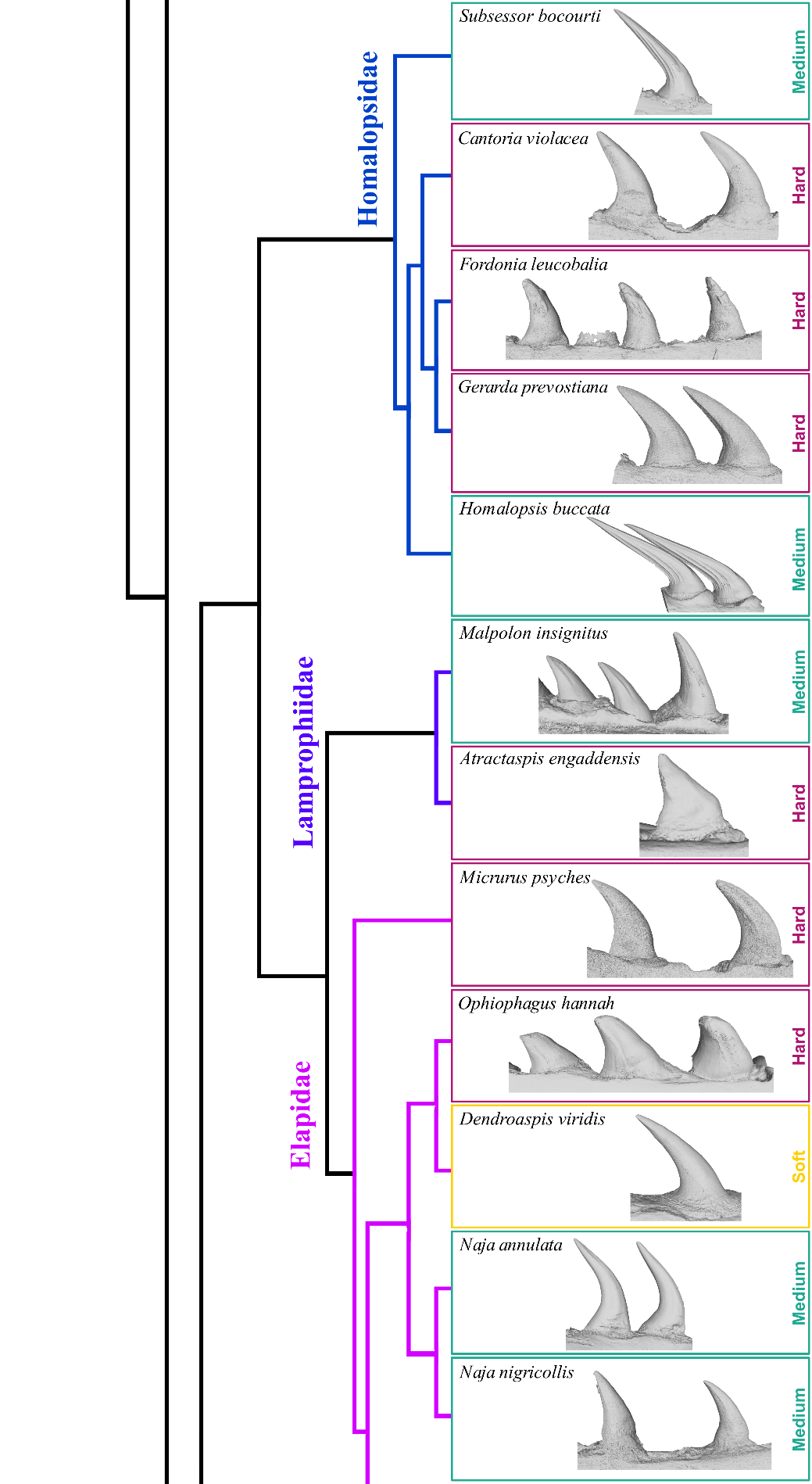


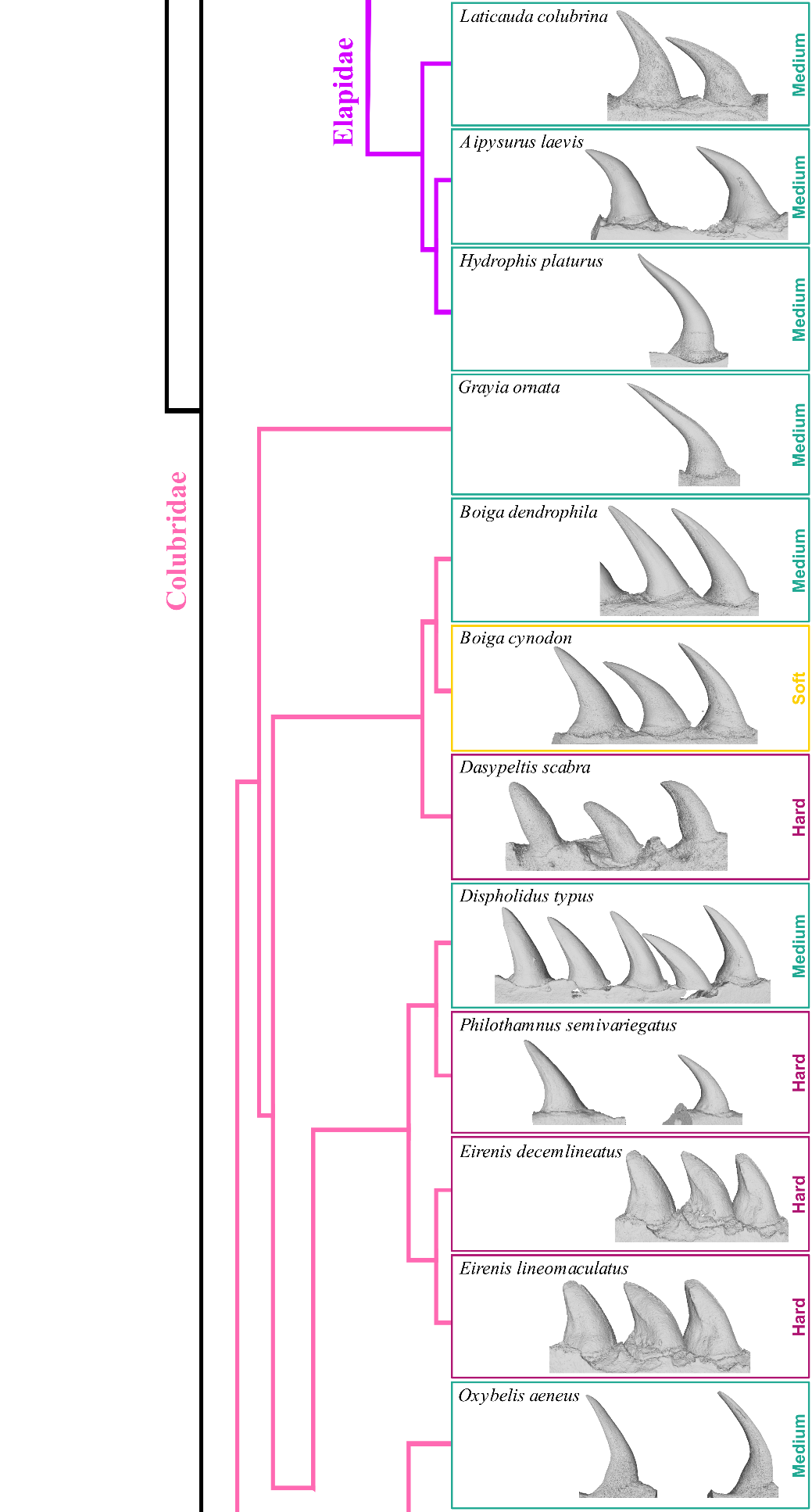


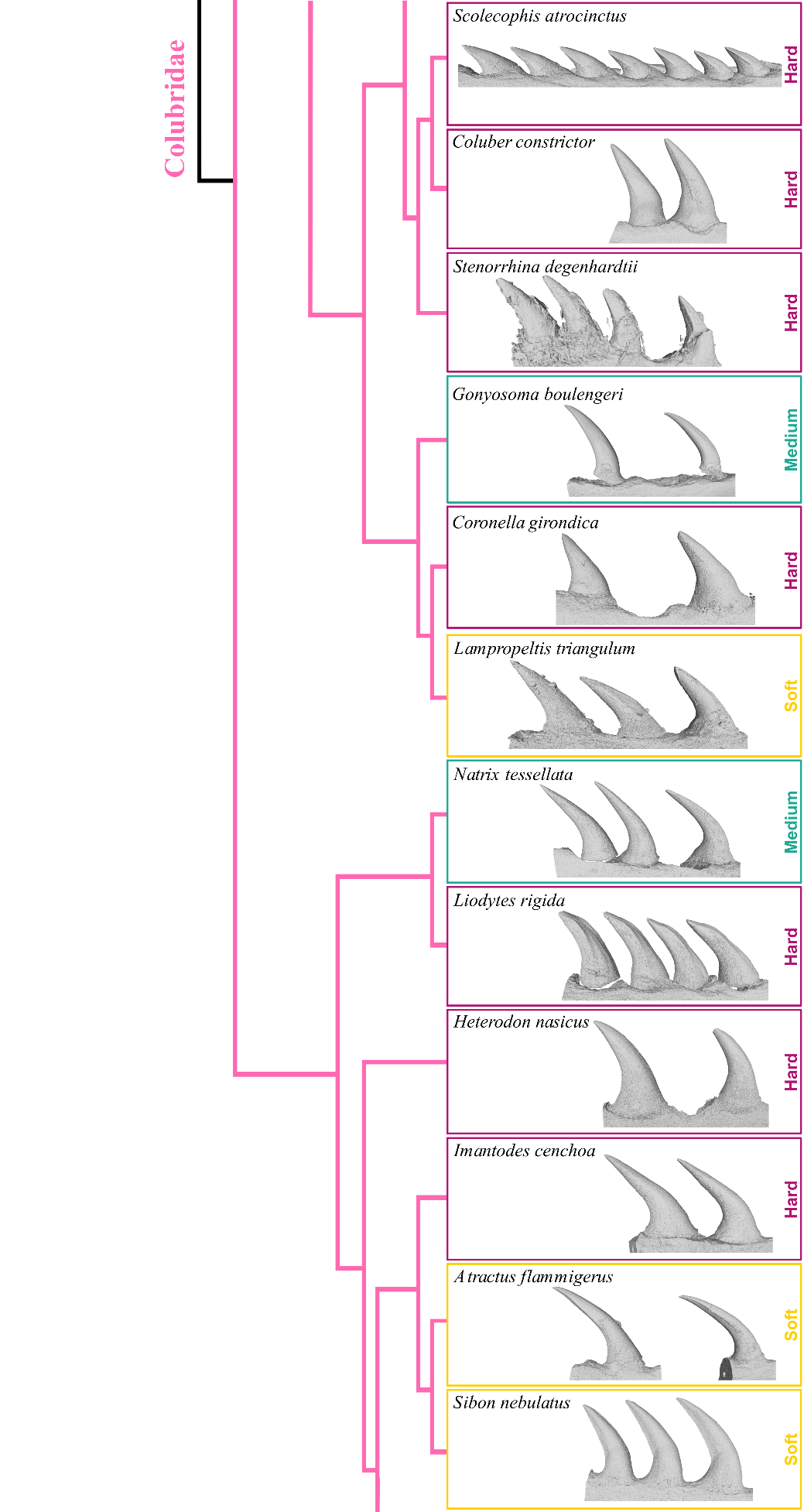


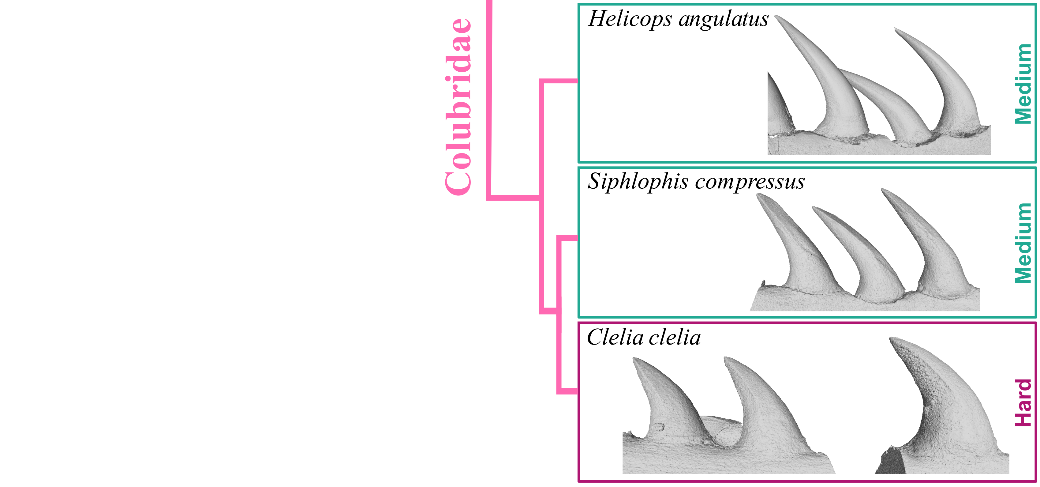


**Supplementary Material 3:** Description of anatomical landmarks placed on the inner (blue) and outer (red) layers of the teeth. Left figure: medial view of the tooth. Right picture: posterior view of the tooth.

| **LM#** | **Name** |
| --- | --- |
| **1** | Tip of the pulp cavity |
| **2** | Most anterior point of the tooth insertion |
| **3** | Most posterior point of the tooth insertion - according to the orientation with the vascular canal |
| **4** | Most medial point of the tooth insertion |
| **5** | Most lateral point of the tooth insertion |
| **6** | Max. curvature - anterior |
| **7** | Max. curvature - posterior |
| **8** | Tip of the tooth |
| **9** | Most anterior point of the tooth insertion |
| **10** | Most posterior point of the tooth insertion - according to the orientation with the vascular canal |
| **11** | Most medial point of the tooth insertion |
| **12** | Most lateral point of the tooth insertion |
| **13** | Max. curvature - anterior |
| **14** | Max. curvature - posterior |


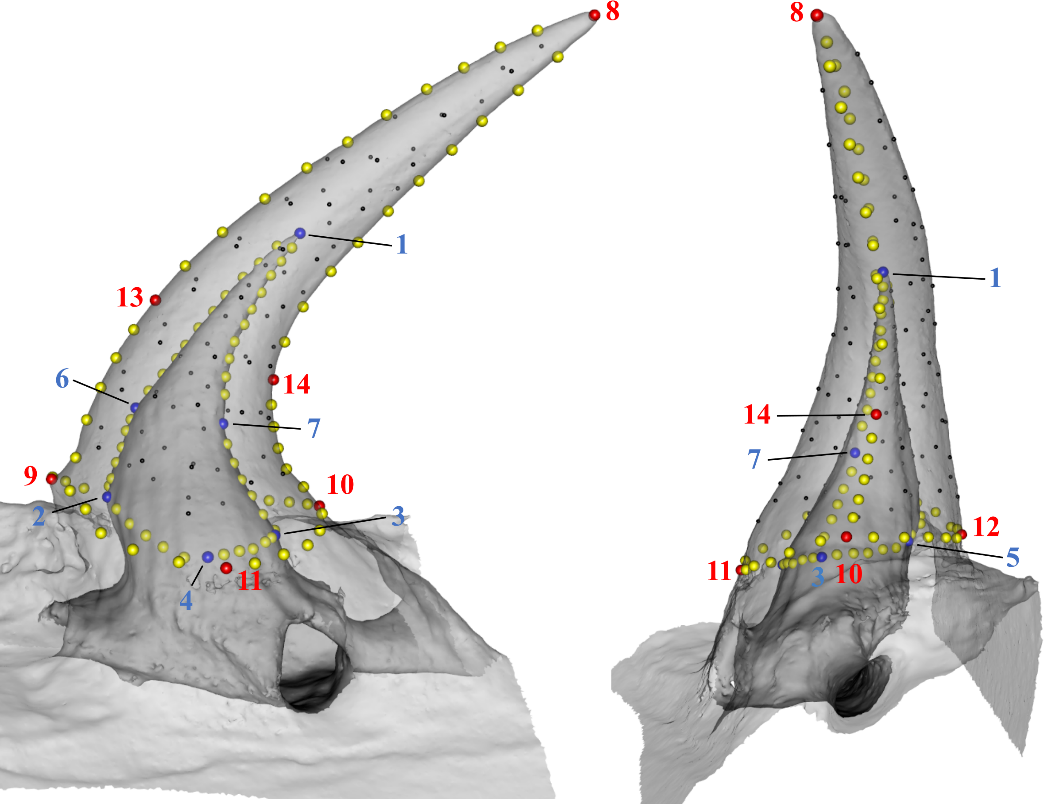


**Supplementary Material 4:** PCA plot showing the repeatability of the anatomical landmark positioning. We landmarked teeth that appear very similar. The plot shows that, despite being similar in shape, repetitions of the same specimen group together and are separated from the other specimens, based on the 14 anatomical landmarks. Yet, some specimens are close (*Candoia carinata* “C_car” and *Boiga cynodon* “B_cyn”), thus suggesting a need for more accurate shape information using curve and surface semi-landmarks.


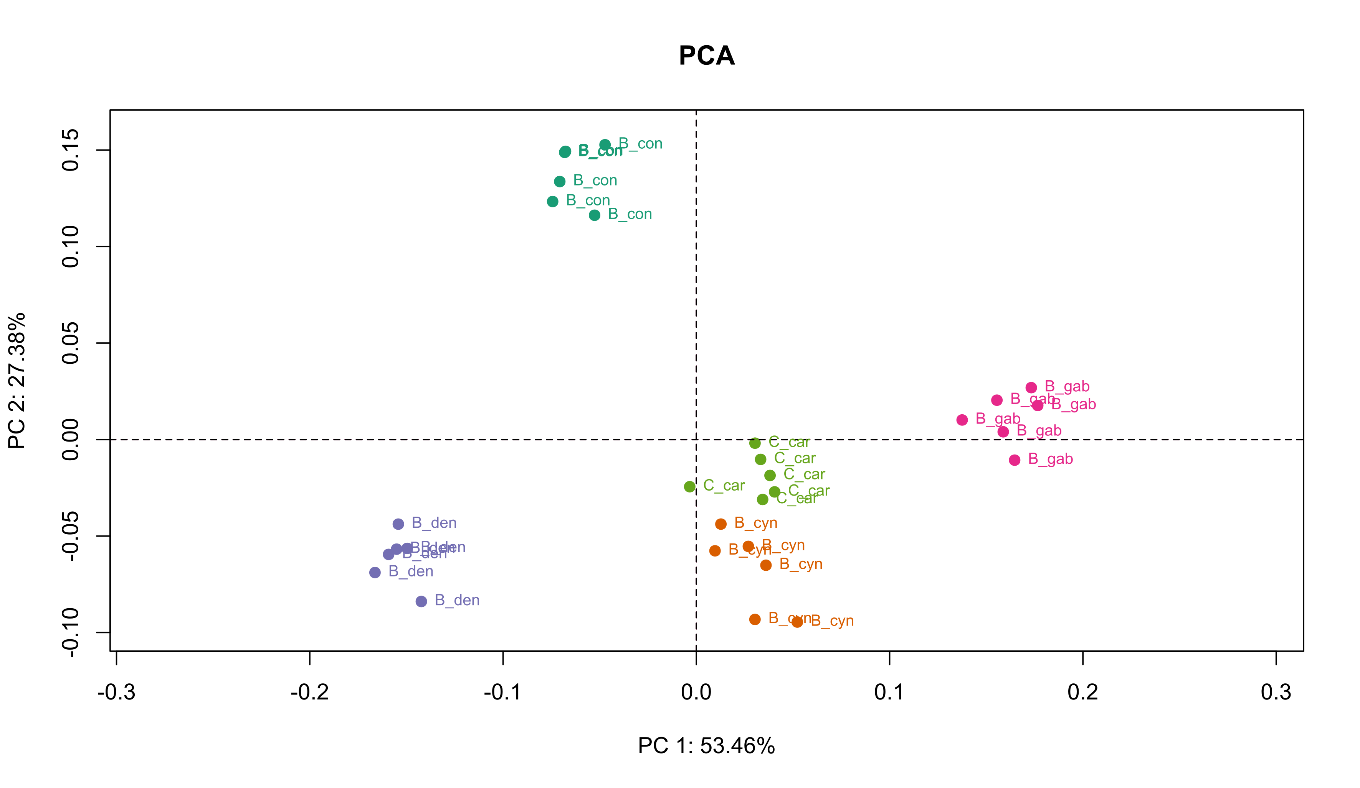


**Supplementary Material 5:** Two examples of curvature digitization using the plugin Kappa in FIJI: *Boa constrictor* (top snapshot), *Eirenis decemlineatus* (bottom snapshot).


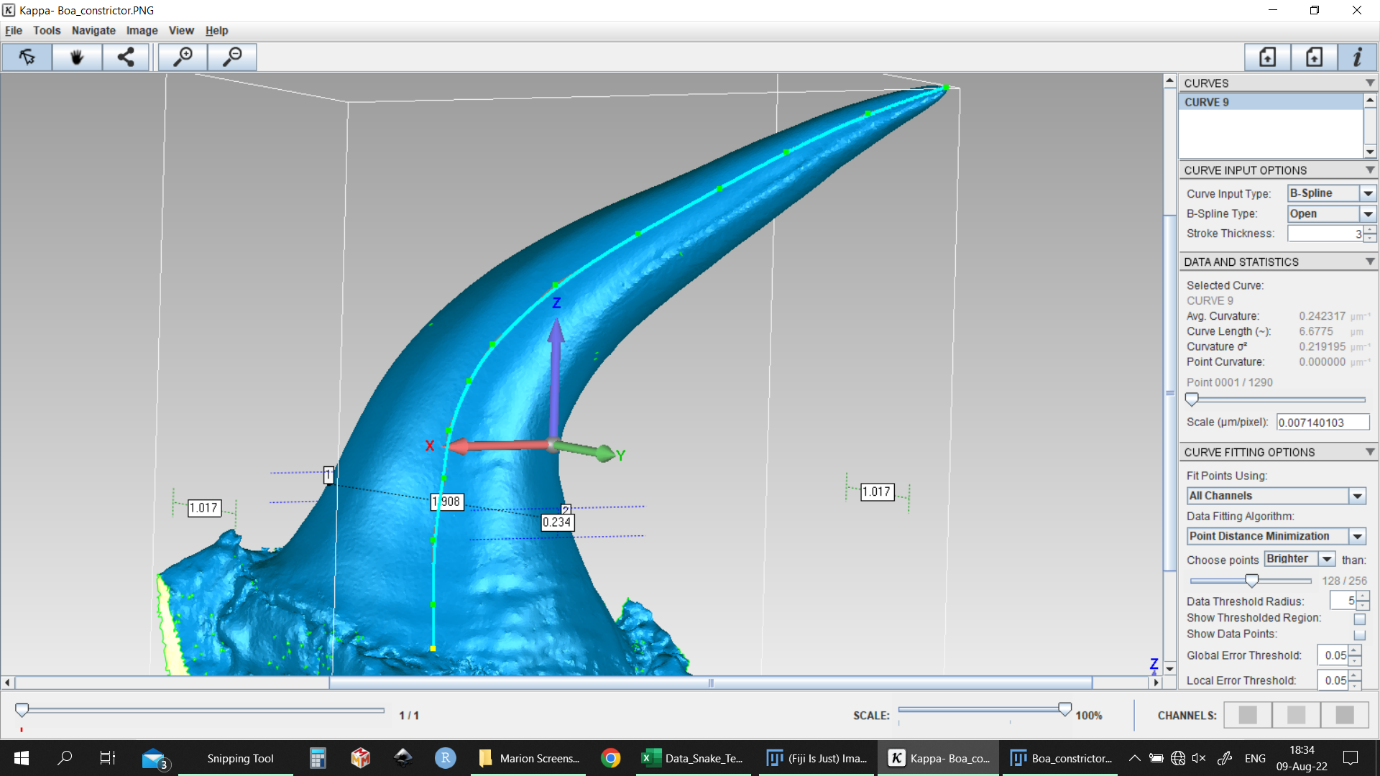


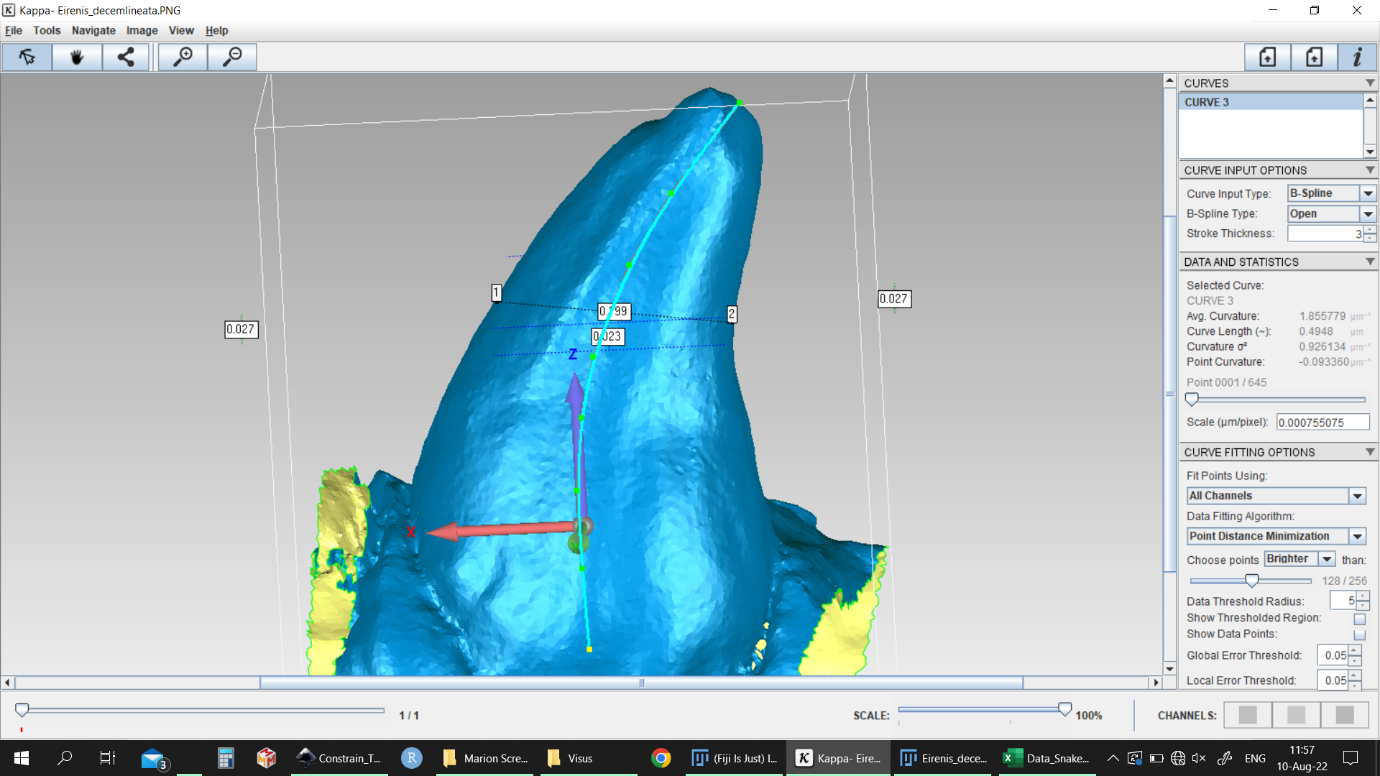


**Supplementary Material 6:** Phylomorphospace of tooth shape variation. Each dot represents one species and is colored according to their main feeding challenge.


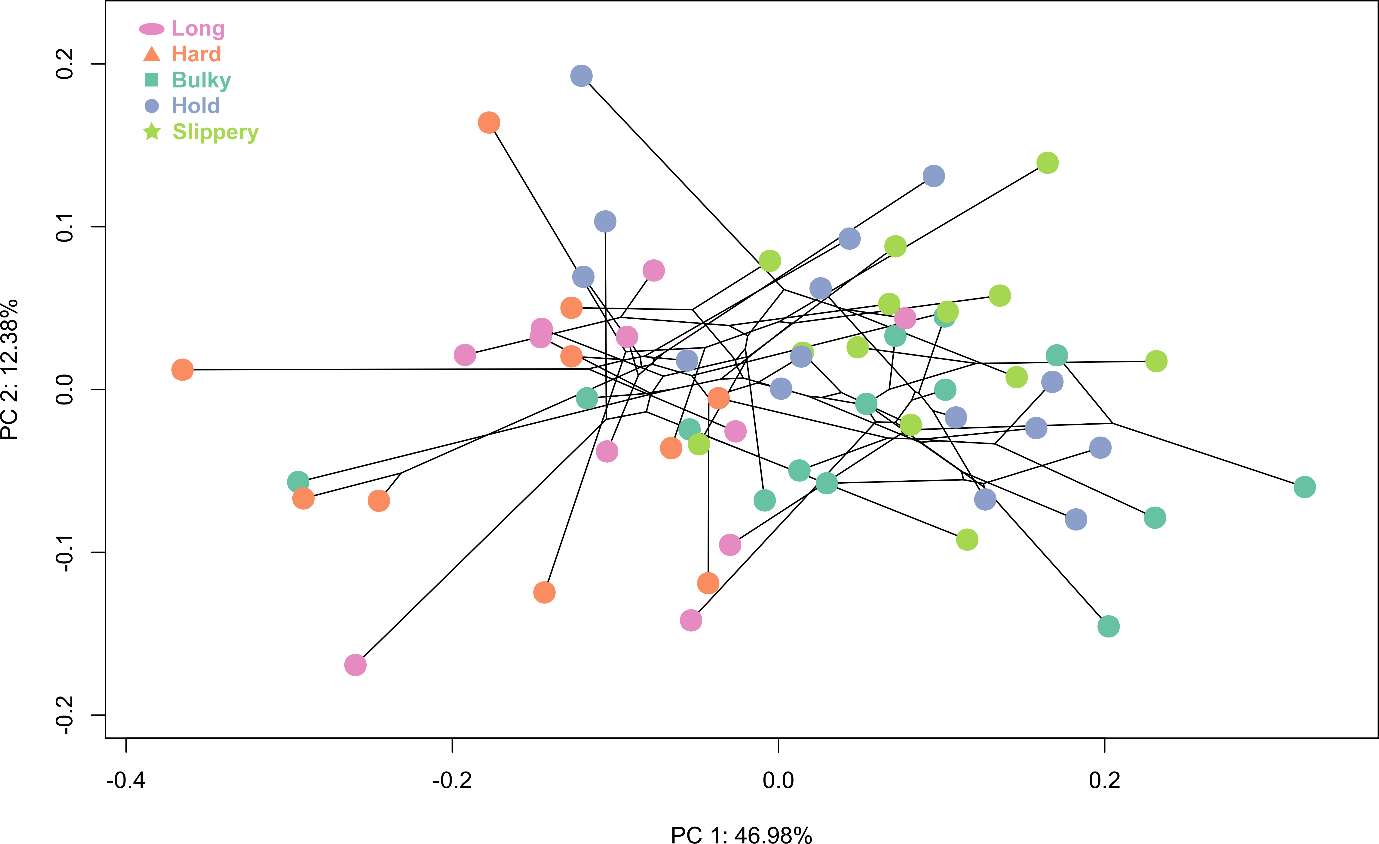


**Supplementary Material 7:** Results of the model fit comparison. Highlighted in grey are models used for post-hoc pairwise tests.

logLik residual.pc.no penalty AIC

Hardness 14562.49 60 4278 -24846.98

Foraging substrate 14515.25 60 4278 -24752.50

Size + Hardness 14305.41 59 4402 -24208.82

Size + Foraging substrate 14266.06 59 4402 -24130.12

Main challenge 13972.51 58 4526 -23419.02

Size + Main challenge 13719.99 57 4650 -22789.98

**Supplementary Material 8:** Eigenvalues, proportion of variance and cumulative proportion of variance associated with each Principal Component.

| Component | Eigenvalues | Proportion of Variance | Cumulative Proportion |
| --- | --- | --- | --- |
| Comp1 | 0.020781618 | 0.470 | 0.470 |
| Comp2 | 0.005475935 | 0.124 | 0.594 |
| Comp3 | 0.003178485 | 0.072 | 0.665 |
| Comp4 | 0.00268192 | 0.061 | 0.726 |
| Comp5 | 0.001979379 | 0.045 | 0.771 |
| Comp6 | 0.001393383 | 0.031 | 0.802 |
| Comp7 | 0.001170302 | 0.026 | 0.829 |
| Comp8 | 0.001005174 | 0.023 | 0.851 |
| Comp9 | 0.000817548 | 0.018 | 0.870 |
| Comp10 | 0.00078064 | 0.018 | 0.888 |
| Comp11 | 0.000621315 | 0.014 | 0.902 |
| Comp12 | 0.000549496 | 0.012 | 0.914 |
| Comp13 | 0.000452438 | 0.010 | 0.924 |
| Comp14 | 0.000414458 | 0.009 | 0.934 |
| Comp15 | 0.000327358 | 0.007 | 0.941 |
| Comp16 | 0.000270363 | 0.006 | 0.947 |
| Comp17 | 0.000234639 | 0.005 | 0.952 |
| Comp18 | 0.000223911 | 0.005 | 0.957 |
| Comp19 | 0.000186986 | 0.004 | 0.962 |
| Comp20 | 0.000163859 | 0.004 | 0.965 |
| Comp21 | 0.000143923 | 0.003 | 0.969 |
| Comp22 | 0.000137272 | 0.003 | 0.972 |
| Comp23 | 0.000121359 | 0.003 | 0.975 |
| Comp24 | 0.000113178 | 0.003 | 0.977 |
| Comp25 | 8.65E-05 | 0.002 | 0.979 |
| Comp26 | 8.51E-05 | 0.002 | 0.981 |
| Comp27 | 8.20E-05 | 0.002 | 0.983 |
| Comp28 | 7.35E-05 | 0.002 | 0.984 |
| Comp29 | 7.24E-05 | 0.002 | 0.986 |
| Comp30 | 6.03E-05 | 0.001 | 0.987 |
| Comp31 | 4.78E-05 | 0.001 | 0.989 |
| Comp32 | 4.67E-05 | 0.001 | 0.990 |
| Comp33 | 4.03E-05 | 0.001 | 0.991 |
| Comp34 | 3.66E-05 | 0.001 | 0.991 |
| Comp35 | 3.48E-05 | 0.001 | 0.992 |
| Comp36 | 3.30E-05 | 0.001 | 0.993 |
| Comp37 | 3.02E-05 | 0.001 | 0.994 |
| Comp38 | 2.65E-05 | 0.001 | 0.994 |
| Comp39 | 2.36E-05 | 0.001 | 0.995 |
| Comp40 | 2.32E-05 | 0.001 | 0.995 |
| Comp41 | 2.01E-05 | 0.000 | 0.996 |
| Comp42 | 1.91E-05 | 0.000 | 0.996 |
| Comp43 | 1.56E-05 | 0.000 | 0.996 |
| Comp44 | 1.47E-05 | 0.000 | 0.997 |
| Comp45 | 1.41E-05 | 0.000 | 0.997 |
| Comp46 | 1.33E-05 | 0.000 | 0.997 |
| Comp47 | 1.31E-05 | 0.000 | 0.998 |
| Comp48 | 1.17E-05 | 0.000 | 0.998 |
| Comp49 | 1.07E-05 | 0.000 | 0.998 |
| Comp50 | 1.01E-05 | 0.000 | 0.998 |
| Comp51 | 9.28E-06 | 0.000 | 0.999 |
| Comp52 | 8.16E-06 | 0.000 | 0.999 |
| Comp53 | 7.93E-06 | 0.000 | 0.999 |
| Comp54 | 6.49E-06 | 0.000 | 0.999 |
| Comp55 | 6.16E-06 | 0.000 | 0.999 |
| Comp56 | 5.91E-06 | 0.000 | 0.999 |
| Comp57 | 5.41E-06 | 0.000 | 1.000 |
| Comp58 | 4.95E-06 | 0.000 | 1.000 |
| Comp59 | 4.30E-06 | 0.000 | 1.000 |
| Comp60 | 4.01E-06 | 0.000 | 1.000 |
| Comp61 | 3.58E-06 | 0.000 | 1.000 |
| Comp62 | 3.16E-06 | 0.000 | 1.000 |
